# Evaluation of the Effects of Sumac (*Rhus coriaria*) Extract-Loaded Ethosomes on an *In Vitro* Wound Healing Model

**DOI:** 10.1021/acsomega.5c00910

**Published:** 2025-06-18

**Authors:** Melis Emanet, Matteo Battaglini, Alessio Carmignani, Federico Catalano, Martina Bartolucci, Andrea Petretto, Gianni Ciofani

**Affiliations:** † Smart Bio-Interfaces, Istituto Italiano di Tecnologia, Viale Rinaldo Piaggio 34, 56025 Pontedera, Italy; ‡ Electron Microscopy Facility, Istituto Italiano di Tecnologia, Via Morego 30, 16163 Genova, Italy; § Clinical Proteomics and Metabolomics Core Facility, IRCCS, Istituto Giannina Gaslini, Via Gerolamo Gaslini 5, 16147 Genova, Italy

## Abstract

Wound healing involves a series of complex bioprocesses, including repairing skin damage, maintaining its barrier features, and preserving all other skin functions. Since the skin is the primary organ exposed to external factors, these bioprocesses can be interrupted by potential exogenous toxicants. Efforts to mitigate the effects of these toxicants can help accelerate the healing process, facilitating complete wound recovery. In this context, sumac (*Rhus coriaria*) extract, rich in polyphenolics with antioxidant and anti-inflammatory properties, can be exploited to overcome oxidant and inflammation-dependent burdens. Ethosomes, lipid-based intradermal delivery vehicles, have been selected for the delivery of sumac extract, as they enhance penetration through the skin layers. Considering their remarkable flexibility and deformability, ethosomes can minimize drug leakage even under harmful penetration conditions. Given the diverse bioactive content of sumac extract, ethosomes have been considered ideal for delivering both hydrophilic and lipophilic active compounds. Sumac extract (SuExt)-loaded ethosomes (SuExt-ethosomes) were therefore produced and characterized. These nanocarriers demonstrated significant cellular internalization and cytocompatibility in human dermal fibroblasts (HDFs), along with excellent antioxidant and anti-inflammatory activity. A comprehensive investigation, supported by proteomic analysis, revealed that SuExt-ethosomes present promising wound healing potential, supporting future investigations in preclinical models.

## Graphical abstract

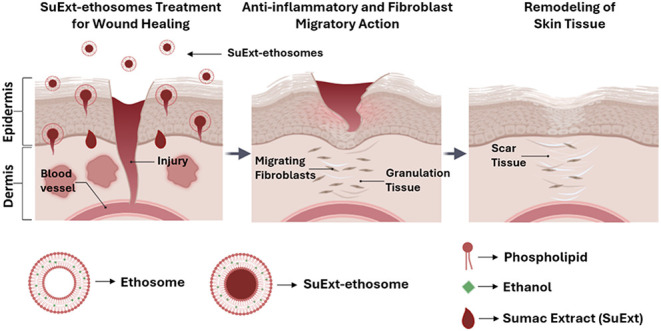

## 1 Introduction

Wound healing consists of four overlapping biological processes: homeostasis, inflammation, proliferation, and remodeling, all of which aim to restore skin integrity.^1^ The progression of each phase varies depending on the wound characteristics, in particular, in terms of the potential contamination or infection by external toxicants and microorganisms. During the initial homeostasis phase, platelet activation and fibrin production lead to platelet aggregation, which serves as a barrier against the disruption of vessel integrity.^2^ These platelet aggregates stimulate the inflammatory response by secreting a wide variety of cytokines such as platelet-derived growth factor (PDGF), transforming growth factor (TGF), epidermal growth factor (EGF), and insulin growth factor (IGF). These cytokines promote the maturation of monocyte-derived macrophages and guide them via chemotaxis to phagocyte cellular debris and microorganisms.^3^ Additionally, cytokines induce the proliferation and migration of fibroblasts and keratinocytes across the wound area to help recreate the skin layer.^4^ Eventually, epidermal cells express collagen and matrix metalloproteinase, which facilitate tissue remodeling.^5^ External factors, such as microorganisms and toxicants, which are the primary skin antagonists, can potentially disrupt these biological pathways, leading to complications in the healing process.

Natural extracts, derived from plants, herbs, and other vegetal sources, are rich in phytochemicals, compounds exhibiting several different biological activities.^6^ Flavonoids, phenolic acids, tannins, stilbenes, lignans, coumarins, essential fatty acids, and nonflavonoids such as alkaloids and terpenoids not only play a significant role in the plant defense mechanisms but also provide a wide range of health benefits to humans. Research has shown, for example, that these extracts possess antioxidant, anti-inflammatory, antimicrobial, anticancer, and immunomodulatory properties.^7^ The antioxidant activity of phytochemicals is mainly due to the presence of peculiar chemical groups, such as hydroxyl, in their molecular structure, which scavenge reactive oxygen species and contribute to inhibiting inflammatory processes.^8^ For example, flavonoids like quercetin are known for their ability to neutralize free radicals, while terpenoids such as curcumin have demonstrated anti-inflammatory effects.^9^ Furthermore, phytochemicals play a key role in antimicrobial activity by altering the bacterial membrane fluidity.^10^ The biological activities of these compounds make natural extracts a promising source of therapeutic agents, offering potential applications in medicine, cosmetics, and nutrition. Their relatively low toxicity further adds to their appeal as alternative or complementary therapies in modern healthcare.

Sumac (*Rhus coriaria*) extract (SuExt), an example of a natural active substance mixture, has long been exploited for treating skin irritations, wounds, and infections in traditional medicine in the Middle East and the Mediterranean regions. Sumac, a common name for the genus *Rhus*, is composed of 250 individual species, but a few of them have been investigated for their potential pharmaceutical properties. *Rhus coriaria*, one of the most well-known species, grows in the Canary Islands, Iran, Afghanistan, and southeast Turkey and is commonly used as a food spice. Previous phytochemical investigations of *Rhus coriaria* have identified a diverse array of bioactive compounds, including gallic acid, methyl gallate, quercetin, kaempferol, tannins, and anthocyanins.^11^ These compounds are well-known for their antioxidant, anti-inflammatory, antimicrobial, and tissue-repairing properties, all of which are crucial in the context of wound healing.^12^ For example, gallic acid and methyl gallate have demonstrated strong free radical scavenging activity and anti-inflammatory potential by modulating oxidative stress pathways and cytokine expression.^13^ Flavonoids such as quercetin and kaempferol are known to stimulate fibroblast proliferation, enhance collagen synthesis, and reduce inflammation, all essential steps in the wound healing process.^14^ The presence of high-molecular-weight hydrolyzable tannins also contributes to the astringent and antimicrobial effects traditionally associated with sumac.^15^ A study revealed the ability of sumac to modulate collagen and glycosaminoglycan production, which are critical components in tissue remodeling during wound healing.^16^ Furthermore, recent *in vitro* studies, including our previous works, demonstrated that sumac extract effectively protects human dermal fibroblasts from oxidative stress-induced damage.^17^

The bioactive compounds present in SuExt include phenolics, such as betaine, choline, trigonelline, and various flavonoids, which provide a solid rationale for exploring their effects on skin regeneration processes. Sumac-derived bioactive compounds are, however, particularly sensitive to environmental conditions and thus require a delivery system capable of both protecting and targeting them to the intended site. Given their affinity for molecules characterized by highly heterogeneous physical/chemical properties, ethosomes represent ideal candidates as delivery agents for SuExt.

Ethosome-based systems are characterized by the presence of alcohol, in general ethanol, which remains in the vesicles even after the synthesis process and confers high flexibility and stability to the nanostructures, facilitating their penetration into the skin.^18^ Their high flexibility also implies a reduced fragility, a decreased drug leakage, and thus a higher amount of drug reaching the therapeutic site. This feature also results in a higher encapsulation extent, improving drug delivery efficiency in terms of payload targeting.^19^ Furthermore, ethanol reduces lipid density following a phenomenon known as “lipid fluidization”, which decreases the compact arrangement of the skin outer lipid layers and of the cell membranes, facilitating the penetration of vesicles through the skin layers.^20^ Ethosomes’ high affinity to both hydrophobic and hydrophilic molecules makes them an effective delivery system, especially when dealing with a mixture of highly heterogeneous compounds, like in the case of SuExt.^21^

In this study, we prepared and characterized sumac extract-loaded ethosomes (SuExt-ethosomes) and tested their activity on human dermal fibroblasts (HDFs). Cellular internalization was confirmed through confocal microscopy images and flow cytometry analysis; the effect of SuExt-ethosomes on cell migration was evaluated by observing the scratch closure in HDF cultures treated with nanoparticles over time. SuExt-ethosomes were also assessed for their protective effects on insulted cultures; eventually, their impact on HDFs in the wound healing process was comprehensively investigated through proteomic analysis.

## 2 Materials and Methods

### 2.1 Extraction of Sumac Active Compounds

Sumac samples (*Rhus coriaria* from Gaziantep, Guneydogu, Turkey) were obtained from local producers. The extraction of active compounds was performed following the protocol described in a previous study of our group.^22^ To ensure reproducibility and concentration of the active compounds, the sumac samples were freeze-dried for 24 h just after freezing at −80 °C for 24 h; hydroethanolic extraction was followed by dispersing 6 g of freeze-dried sumac powder in 40 mL of ethanol aqueous solution (1:1 v/v) and continuously stirring at 240 rpm on a shaker at 20 °C overnight. The extract mixture was filtered using a Whatman grade 1 paper filter to eliminate solid residues; for further purification, the mixture was centrifuged three times at 8000*g* for 10 min. Eventually, the resulting supernatant was collected and stored at −20 °C in the dark for further experiments.

### 2.2 Fabrication and Characterization of Ethosomes and SuExt-Ethosomes

#### 2.2.1 Fabrication of Ethosomes and SuExt-Ethosomes

To synthesize ethosome, two separate suspensions, one in an organic phase and the other in a hydrophilic phase, were mixed under controlled conditions. The organic phase was composed of s-phosphatidylcholine (190 mg), cholesterol (10 mg), and ethanol (3 mL) and sonicated in a sonication bath for 2 min to ensure optimal dissolution. This suspension was then constantly stirred at 600 rpm at 30 °C, while the hydrophilic solution (7 mL of dH_2_O) was gradually added at a constant velocity under controlled environmental conditions. In the SuExt-ethosome production, 1.5 mL of SuExt (24 mg/mL) was diluted in 5.5 mL of H_2_O. For a uniform nanoparticle size, the final suspension was sonicated using a tip sonicator (Fisherbrand Q125 Tip Sonicator) at 90% amplitude for 4 min. Afterward, the samples were cooled down by storing at 4 °C for 1 h; a homogenization step followed, carried out with a high-pressure homogenizer (20 psi homogenizing pressure). The obtained ethosomes and SuExt-ethosomes were first filtered with a 0.45 μm filter to eliminate big aggregates and then with a 0.2 μm filter (Sartorius Minisart Plus Syringe Filters) for further purification. A final purification was performed using an Amicon Ultra-4 centrifugal filter (100 kDa, Sigma-Aldrich) at 5000 rpm for 5 min, repeated three times, to remove nonloaded extracts and free lipids. Eventually, the final samples were stored in glass vials in the dark at 4 °C for further experiments. To assess the production yield, three aliquots of ethosome and SuExt-ethosome dispersions (50 μL) were first frozen at −20 °C for 12 h and then freeze-dried overnight. The dried mass was weighed, and the average mass of the three aliquots for each sample was assessed. By comparing this value to the sample mass at the beginning of the reaction, the production yield was determined.

For fluorescence tracking, both ethosomes and SuExt-ethosomes (500 μg/mL) were stained with the Vybrant DiO dye (5 μM, Invitrogen) by stirring the dispersion at 150 rpm for 16 h at room temperature. A washing step was performed by filtering with an Amicon Ultra-4 centrifugal filter in dH_2_O at 800*g* for 5 min.

#### 2.2.2 Characterization of Ethosomes and SuExt-Ethosomes

The morphological analysis was conducted using transmission electron microscopy (TEM) with a JEOL JEM1011 transmission electron microscope equipped with a thermionic electron source (tungsten) and operating at 100 kV. A drop of the sample dispersion was placed on a Cu grid coated with ultrathin amorphous carbon film, which was first plasma treated (O_2_ + Ar plasma, 10 W, 2 min) to remove hydrocarbon residues from carbon film deposition. To enhance the contrast of the lipid coating of the particles, a negative staining procedure was applied using a uranyl acetate solution (1% v/v) for 60 s.

The colloidal stability was assessed by measuring hydrodynamic size distribution, polydispersity index (PDI), and ζ potential using ZetaSizer (Malvern Instruments). For these measurements, 1 mL of samples (50 μg/mL) was dispersed in deionized water and each sample was measured three times at room temperature. Long-term stability has been evaluated as well by storing the nanoparticles in dH_2_O at 4 °C up to 28 days.

Raman spectra were obtained using a Renishaw In Via Reflex Raman microscope (Renishaw) equipped with a 514 nm argon ion laser. A minimum of 16 spectra were acquired from different regions of the dried samples.

The SuExt encapsulation efficiency (EE) and loading capacity (LC) in ethosomes were determined by using high-pressure liquid chromatography (HPLC, Shimadzu LC-20A). At this aim, upon freeze-drying, SuExt-ethosomes were dissolved in methanol/water (1:1 v/v) and heated at 75 °C for 2 h under stirring. Then, the lipid component has been removed by centrifugation at 37,000*g* at 4 °C for 1 h. The supernatant was assessed by HPLC by measuring absorbance values of one of the most reproducible peaks present in the mixture (Figure S1A) and comparing them with those of SuExt samples of known concentrations (0, 0.075, 0.15, and 0.3 μg/mL; Figure S1B). The following parameters have been adopted: flow rate of 1.0 mL/min, detection wavelength of 365 nm, injection volume of 5 μL, and run time of 20 min. The mobile phase consisted of solvent A (water) and solvent B (acetonitrile), with the following gradient program: 0–5 min, 20% B; 5–10 min, 40% B; 10–15 min, 50% B; 15–20 min, 20% B; followed by re-equilibration to initial conditions. EE and LC have been eventually calculated according to, respectively, eqs 1 and 2:

1 $$EE\;\left(\%\right)=W_{loaded}/W_{total}\times 100$$

2 $$\mathrm{LC}\;\left(\%\right)=W_{\mathrm{loaded}}/W_{\mathrm{freeze}‐\mathrm{dried}}\times 100$$

where *W*_total_ is the total amount of SuExt (in mg) used in the preparation, *W*_loaded_ is the SuExt amount (in mg) loaded in the ethosomes assessed through HPLC, and *W*_freeze‑dried_ is the freeze-dried amount (in mg) of SuExt-ethosomes.

#### 2.2.3 Phenolic Content and Total Antioxidant Capacity Determination

The total phenolic content of SuExt-ethosomes was evaluated by using the Folin-Ciocalteu reagent assay (Sigma- Aldrich), which determines the total phenolic content relative to a standard compound (tannic acid at increasing concentrations: 0, 25, 50, 100, 150, 250, 500, and 1000 μg/mL). The assay was performed by dispersing 100 μL of Folin-Ciocalteu reagent, 300 μL of sodium carbonate (20% w/v in water), and 20 μL of SuExt-ethosomes (100 μg/mL) or tannic acid in 1580 μL of dH_2_O in 24-well plates. The samples were incubated at 37 °C for 35 min, and the absorbance was measured using a VictorX3 microplate reader at 760 nm. The tannic acid-equivalent phenolic content was calculated based on the standard curve generated from tannic acid concentrations.

The total antioxidant capacity of SuExt-ethosomes was assessed using a total antioxidant capacity detection kit (Sigma-Aldrich) following the manufacturer’s instructions. SuExt-ethosomes (50 μL; 100 μg/mL) or the standard compound Trolox (50 μL; 0, 80, 120, 160, 200, and 400 μM) was mixed with a Cu^2+^-containing buffer solution (100 μL) and incubated in the dark at room temperature for 90 min. The absorbance of the samples was then measured at 570 nm using the microplate reader, and the antioxidant capacity of the samples was calculated as the Trolox equivalent, according to the obtained standard curve.

### 2.3 Cellular Studies

#### 2.3.1 Cell Cultures

Human dermal fibroblasts (HDFs) have been obtained from ATCC (PCS-201-012) and cultured in high-glucose Dulbecco’s modified Eagle’s Medium (DMEM high glucose, EuroClone) supplemented with 10% heat-inactivated fetal bovine serum (FBS), 1% sodium pyruvate (1 mM, EuroClone), and 1% penicillin-streptomycin solution (100 U/mL of penicillin and 100 μg/mL of streptomycin, EuroClone). The cell culture was maintained at 37 °C in a 5% CO_2_ humidified atmosphere. The experiments were performed on cells within 10–20 passages.

#### 2.3.2 Cytocompatibility Assessment

The metabolic activity of the HDFs following treatment with nanoparticles was assessed using a WST-1 colorimetric assay (BioVision), conducted according to the manufacturer’s instructions. Cells were seeded at a density of 1 × 10^4^ cells/cm^2^ in 96-well plates and incubated overnight. The cultures were then treated with ethosomes, SuExt-ethosomes (0, 10, 25, 50, 100, 250, 500, and 1000 μg/mL), and SuExt (0, 1.4, 3.4, 6.8, 13.6, 34, 68, and 136 μg/mL; corresponding to the content loaded in 0, 10, 25, 50, 100, 250, 500, and 1000 μg/mL of SuExt-ethosomes) and further incubated for 24 h. At the endpoint, the medium was replaced with the WST-1 reagent dissolved in DMEM (Gibco) without phenol red (10% v/v), and the cells were incubated at 37 °C for 45 min. Eventually, the supernatants were collected, and the absorbance was measured at 440 nm by using the plate reader; experiments were performed in triplicate.

Cellular proliferation following nanoparticle treatment was analyzed using the Quant-iT PicoGreen dsDNA assay kit (Invitrogen) according to the manufacturer’s instructions. The cells were processed as described for metabolic activity assessment and thereafter rinsed with PBS three times and left under 150 μL of Milli-Q water, followed by three freezing/thawing cycles between −80 °C and room temperature to induce complete cell lysis. The cell lysates (50 μL) were dispersed in 100 μL of reaction buffer and 150 μL of PicoGreen reagent; after 10 min incubation at room temperature, fluorescence emission (directly proportional to the dsDNA content and thus to the cell number) was measured using the microplate reader (λ_ex_ = 485 nm, λ_em_ = 535 nm); experiments were performed in triplicate.

#### 2.3.3 Cellular Internalization

For confocal microscopy imaging, HDFs were seeded at a 1 × 10^4^ cells/cm^2^ density in μ-Dishes (35 mm, Ibidi) and incubated for 24 h. The cultures were then incubated with DiO-labeled SuExt-ethosomes (100 μg/mL) for further 24 h. Following the treatment, the cells were fixed with 4% paraformaldehyde (PFA, Sigma-Aldrich) at 4 °C for 20 min and then rinsed three times with PBS (Sigma-Aldrich). Next, the cells were treated at 37 °C for 45 min with Hoechst 33342 (1:1000 v/v, Invitrogen) for nuclei visualization and with TRITC-phalloidin (1:200 v/v, Sigma-Aldrich) for *f*-actin staining. 2D images and 3D rendering were acquired with a confocal fluorescence microscope (C2 system, Nikon).

Cellular internalization of DiO-stained SuExt-ethosomes was further quantified through flow cytometry. After the treatment, the cells were collected and resuspended in PBS (pH 7.4) for analysis using a Beckman Coulter CytoFLEX instrument (λ_ex_ = 488 nm, λ_em_ = 530 nm).

#### 2.3.4 Scratch Assay

The migration capacity of HDFs following the treatment with the nanoparticles was assessed using a scratch assay as previously reported.^23^ HDFs were seeded at a density of 3 × 10^4^ cells/well in a 6-well plate. To synchronize proliferation, the cultures were first incubated in a serum-free medium for 24 h, followed by an additional 24 h incubation in the complete medium at 37 °C. Once the cells reached confluence, a sterile pipette tip was used to create a linear scratch in the monolayer to simulate a wound area, and the cultures were rinsed with PBS to remove debris. Afterward, the cells were treated with ethosomes (100 μg/mL), SuExt-ethosomes (100 μg/mL), or SuExt (13.6 μg/mL, corresponding to the content loaded in 100 μg/mL of SuExt-ethosomes) for a further 48 h at 37 °C. Images of the scratches were captured with a Zeiss PrimoVert fluorescence microscope at 0, 12, and 24 h just after staining with calcein (1 μM, Sigma-Aldrich, for 15 min) for fluorescence visualization; the scratch closure was quantified using ImageJ software, and the wound healing effect of the nanoparticles was evaluated by comparing the results to control cultures treated only with plain medium.

#### 2.3.5 Reactive Oxygen Species Detection

HDFs were seeded at a density of 1 × 10^4^ cells/cm^2^ in 24-well plates, and the oxidative stress level following the different treatments was analyzed. Intracellular ROS production was induced by incubating the cultures with *tert*-butyl hydroperoxide (tBH, 200 μM, Sigma-Aldrich) for 24 h. Then, the cells were treated with ethosomes (100 μg/mL), SuExt-ethosomes (100 μg/mL), or SuExt (13.6 μg/mL, corresponding to the content loaded in 100 μg/mL of SuExt-ethosomes) for a further 24 h at 37 °C. Both tBH-insulted and noninsulted cultures underwent the already described treatments. At the endpoint, HDFs were stained with 5 μM CellROX Green Reagent (Invitrogen) in PBS for 30 min at 37 °C and analyzed via flow cytometry (λ_ex_ = 498 nm, λ_em_ = 522 nm).

#### 2.3.6 Inflammation Marker Assessment

Anti-inflammatory potentialities of the proposed nanovectors have been evaluated in terms of CD40 marker expression upon treatment with lipopolysaccharide (LPS, 10 μg/mL, Sigma-Aldrich) for 24 h at 37 °C. After incubation, both LPS-insulted and noninsulted cultures were treated with ethosomes (100 μg/mL), SuExt-ethosomes (100 μg/mL), or SuExt (13.6 μg/mL, corresponding to the content loaded in 100 μg/mL of SuExt-ethosomes) for a further 24 h at 37 °C and, at the endpoint, processed with paraformaldehyde (PFA) at 4 °C for 20 min. The cells were then centrifuged at 860*g* for 10 min at 4 °C and thereafter stained with a FITC antihuman CD40 monoclonal antibody (1:100 in PBS, Sigma-Aldrich) by shaking for 60 s at 37 °C. The cells were washed three times, resuspended in PBS, and analyzed via flow cytometry (λ_ex_ = 498 nm, λ_em_ = 522 nm).

#### 2.3.7 Proteomics

For the proteomic analysis, HDFs from the different experimental classes were lysed, reduced, alkylated with the LYSE buffer (50 μL, Preomics) at 95 °C for 10 min, and sonicated (3 cycles, 30 s/cycle) with Ultrasonic Processor UP200 St (Hielscher). Proteins were isolated and digested by the PAC method, automated on a KingFisher Apex robot (Thermo Fisher Scientific) in a 96-well format.^24^ Briefly, the tip plate was stored in plate 1 and lysate samples were stored in plate 2, in a final concentration of 70% acetonitrile and with magnetic beads in a protein/bead ratio of 1:4 (1:1 SpeedBead Magnetic Carboxylate, 45152105050250 and 65152105050250). Washing solutions were in plates 3 and 5 (acetonitrile), plate 6 (70% ethanol), and plate 7 (isopropanol). Plate 8 contained 100 μL of the digestion solution of 25 mM Tris–HCl of pH 8, LysC (Wako) in an enzyme/protein ratio of 1:100 (w/w), and trypsin (Promega) in an enzyme:protein ratio of 1:50. The protein aggregation was carried out in two steps of 1 min of mixing at a medium mixing speed, followed by a 10 min pause each. The sequential washes were performed in 2.5 min with slow speed, without releasing the beads from the magnet. The digestion was set to 2 h at 37 °C at a slow speed. The resulting peptides were analyzed by a nano-UHPLC-MS/MS system using an Ultimate 3000 RSLC equipment coupled to an Orbitrap Q-Exactive Plus mass spectrometer (Thermo Scientific Instrument). Elution was performed with an EASY spray column (75 μm × 50 cm, 2 μm particle size, Thermo Scientific) at a flow rate of 250 nL/min using a linear gradient of 2–45% solution B (80% acetonitrile (ACN), 5% dimethyl sulfoxide (DMSO), 0.1% formic acid (FA) in H_2_O) along 50 min. Orbitrap detection was used for MS1 measurements at a resolving power of 70 000 in a range between 375 and 1500 *m*/*z* with a 3 × 10^6^ automatic gain control (AGC) target and 50 ms maximum injection time (IT). Precursors were selected for data-independent fragmentation with an isolation window width of 34 *m*/*z* and 19 loop count. Higher collisional dissociation (HCD) energy was set to 27%, and MS2 scans were acquired at a resolution of 35 k, 3 × 10^6^ AGC target, and 50 ms maximum IT. All DIA raw files were processed with Spectronaut version 18 using a library-free approach (directDIA) under default settings.^25^ Enzymes/Cleavage Rules were set to Trypsin/P, LysC. The library was generated against the Uniprot Human database (release UP000005640_9606, June 2024). Carbamidomethylation was selected as a fixed modification, and methionine oxidation, N-terminal acetylation, and deamidation (NQ) were selected as variable modifications. False discovery rate (FDR) of peptide-spectrum match (PSM) and peptide/protein groups was set to 0.01. For quantification, Precursor Filtering was set to Identified (Qvalue) and MS2 was chosen as the quantity MS-level.

### 2.4 Statistical Analysis

Student’s *t*-test has been used for comparison between two data sets; for multiple comparisons, analysis of variance (ANOVA) followed by Bonferroni’s *posthoc* test was used. Data are presented as mean value ± standard deviation from three independent experiments; statistical significance was set at *p* < 0.05.

Concerning proteomics, the Protein Quant Pivot Report generated by Spectronaut was statistically evaluated using Perseus software, version 1.6.15.0. Gene ontology (GO) enrichment was obtained with the Web server HumanBase with “skin fibroblast” as background.^26,27^

## 3 Results and Discussion

### 3.1 Ethosome Characterization

Sumac extract has been extensively characterized in a previous work of our group by using a Vanquish Horizon UHPLC coupled to a Q-Exactive Orbitrap mass spectrometer.^18^ Tables S1 and S2 report a semiquantitative evaluation of the compounds present in the extract by using a C18 column in positive and negative ion modes, respectively.

The morphology of bare and SuExt-ethosomes was observed by using TEM imaging (Figure 1A,B, respectively). Ethosomes exhibited irregular shapes and a wide range of sizes, whereas qualitatively, SuExt-ethosomes appear to have a more uniform size distribution and regular spherical shapes. The irregular shape of the ethosomes is likely due to the high deformability of the nanoparticles in their bare form; conversely, the more regular shape of SuExt-ethosomes can be attributed to the reduced elasticity of the nanoparticles due to the presence of the extract. The average hydrodynamic size distribution resulted to be 145.7 ± 45.3 nm for ethosomes and 220.2 ± 12.1 nm for SuExt-ethosomes, values within the optimal nanoscale range (typically 100–300 nm) for enhanced skin permeation and retention in dermal applications (Figure 1C).^28^ The relatively narrow size distribution (PDI < 0.25; Figure 1D) up to 28 days of incubation suggests good homogeneity and stability. The average surface charge (ζ-potential) resulted in −49.8 ± 3.7 mV for ethosomes and −34.2 ± 8.8 mV for SuExt-ethosomes, reflecting a sufficiently high surface charge to confer electrostatic repulsion among vesicles, thus enhancing colloidal stability and preventing aggregation (Figure 1E). The negative charge observed is also consistent with other ethanol-based vesicular systems and is influenced by the presence of phospholipids and plant-derived compounds.^29^

**Figure 1:**
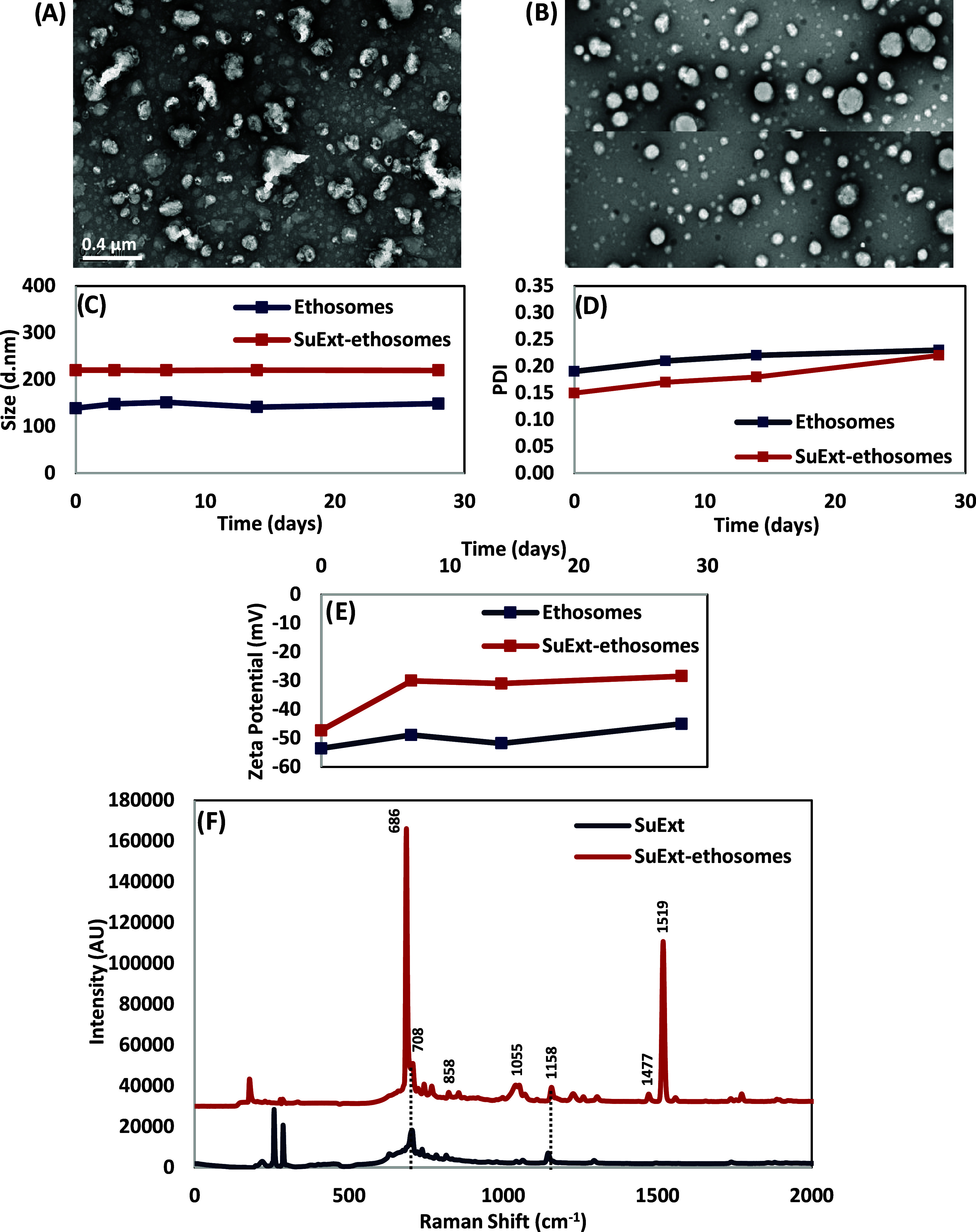
Ethosome characterization. TEM images of plain (A) and SuExt-ethosomes (B). Evaluation of the size distribution (C), PDI (D), and ζ-potential (E) over time. Raman spectroscopy analysis of SuExt and SuExt-ethosomes (F).

The production yield was 96.5 ± 0.1% for ethosomes and 79.0 ± 1.4% for SuExt-ethosomes, while the LC resulted in 13.6 ± 0.1% of total weight. The EE of SuExt in SuExt-ethosome formulation was determined to be 70.4 ± 1.7%, which is considered satisfactory for phytochemical-loaded nanocarriers. This value reflects a successful encapsulation process, particularly given the complex and diverse nature of many compounds present in SuExt. Previous studies have reported similar EE (68.6 ± 1.2%) for plant extract-loaded ethosomes, supporting the effectiveness of our formulation approach.^30^ Collectively, these physicochemical characteristics confirm that the formulation is stable, suitable for dermal application, and in line with expectations for effective skin-targeted delivery systems.

Raman spectroscopy analysis was performed on SuExt and SuExt-ethosomes to give hints about the molecular structures, and the results are reported in Figure 1F. The spectra acquired from SuExt-ethosomes revealed peaks at 858 cm^–1^, attributed to the C=C bending, at 1055 cm^–1^, corresponding to C–C stretching (related to all-trans conformation of the acyl chain), and at 1519 cm^–1^, related to CH_2_ deformation in phosphatidylcholine.^31,32^ Notably, the peaks at 858 and 1519 cm^–1^ were found to be shifted from the original peaks at 874 and 1460 cm^–1^, respectively, demonstrating the chemical interactions of SuExt within the phosphatidylcholine structure.^33^ In addition, peaks at 685 and 1477 cm^–1^ were identified, corresponding to the characteristic CH_2_ deformations of cholesterol. The 685 and 1477 cm^–1^ peaks also shifted from their original positions at 700 and 1451 cm^–1^, suggesting molecular interaction within the cholesterol structure.^34^ Prominent peaks observed in both SuExt and SuExt-ethosomes at 708 and 1158 cm^–1^ were attributed to C–H stretching, typical of the functional groups belonging to the rich molecular components of SuExt. Overall, the observed spectral features confirm the successful loading of SuExt into the nanoparticles.

The specific phenolic content of SuExt was identified through mass spectroscopy analysis in a previous work of our group.^22^ Betaine, the compound in the highest amount, acts as an intracellular osmolyte, regulating cell volume and tissue integrity.^35^ Choline, present at high content as well, is a component of the membrane phospholipids; it plays a multifaceted role in accelerating the wound healing process by supporting cellular repair, reducing inflammation, and protecting against oxidative stress.^36^ Trigonelline is a metabolite that contributes to the stabilization of glucose and lipid levels in the blood, besides having antibacterial effects that play a key role against infections in the wound healing process.^37^

SuExt-ethosomes were characterized in terms of phenolic content, expressed as tannic acid equivalents, and for their antioxidant capacity, expressed as Trolox equivalents. In these tests, SuExt-ethosomes were used at a concentration of 100 μg/mL. The phenolic content was quantified as 5.5 ± 0.6, 15.6 ± 0.4, and 16.15 ± 1.2 ng/mL of tannic acid equivalents for ethosomes, SuExt-ethosomes, and SuExt, respectively. These results indicate that SuExt retains a high level of phenolic compounds, even after encapsulation within the ethosomal carrier. The slight reduction in the phenolic content in the SuExt-ethosome group compared to that of the free extract may be attributed to possible interaction with the lipid bilayers. It is also important to note that ethosomes alone exhibit a minimal but measurable tannic acid-equivalent content, likely due to phospholipid-associated antioxidant properties.

The total antioxidant capacity was found to be 2.8 ± 0.5 ng/mL for ethosomes, 24.2 ± 2.1 ng/mL for SuExt-ethosomes, and 34.4 ± 2.0 ng/mL for SuExt (Trolox equivalents). The total antioxidant capacity was determined using a commercial antioxidant assay kit based on the radical cation decolorization method. This assay operates in water, which is well-suited for detecting water-dispersible ethosomes and water-soluble antioxidants extracted from sumac. The results also show the mild antioxidant properties of the ethosomal phospholipids, and their potential contribution to the observed total antioxidant capacity of SuExt-ethosomes cannot be excluded.

### 3.2 Cytocompatibility and cellular uptake

Cytocompatibility evaluations, performed as described in Section 2.3.2, provided no significant decrement of both cell metabolism (Figure 2A) and proliferation (Figure 2B) following each considered treatment. According to the data collected, a precautionary working concentration of 100 μg/mL SuExt-ethosomes was selected for the following experiments.

**Figure 2:**
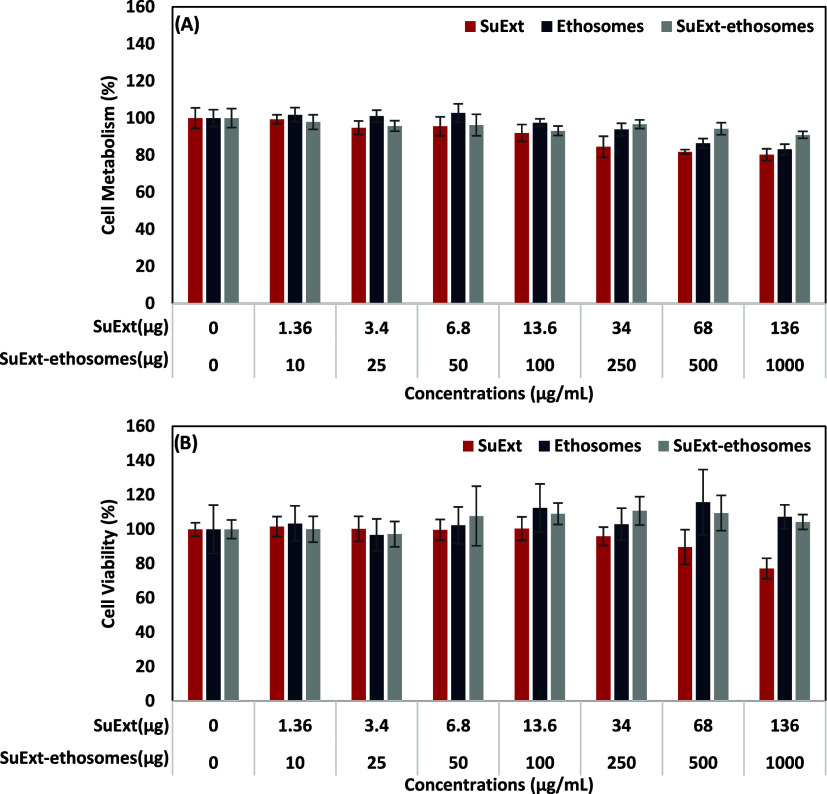
Cytocompatibility evaluation of SuExt, ethosomes, and SuExt-ethosomes at increasing concentrations. WST-1 colorimetric assay, indicative of cell metabolism (A), and PicoGreen dsDNA quantification, indicative of cell viability (B). Data are represented as mean values ± standard deviation (*n* = 6).

Cellular internalization has been qualitatively assessed through confocal microscopy, as reported in Figure 3A (representative single *z*-stacks) and Figure 3B (representative 3D rendering), which show an extensive uptake after a 24 h incubation, with a preferential perinuclear localization of SuExt-ethosomes. Flow cytometry analysis provided a quantitative evaluation of the internalization (Figure 3C; representative fluorescence plots in Figure S2), showing 88.5 ± 0.4% of SuExt-ethosome^+^ cells after the incubation.

**Figure 3:**
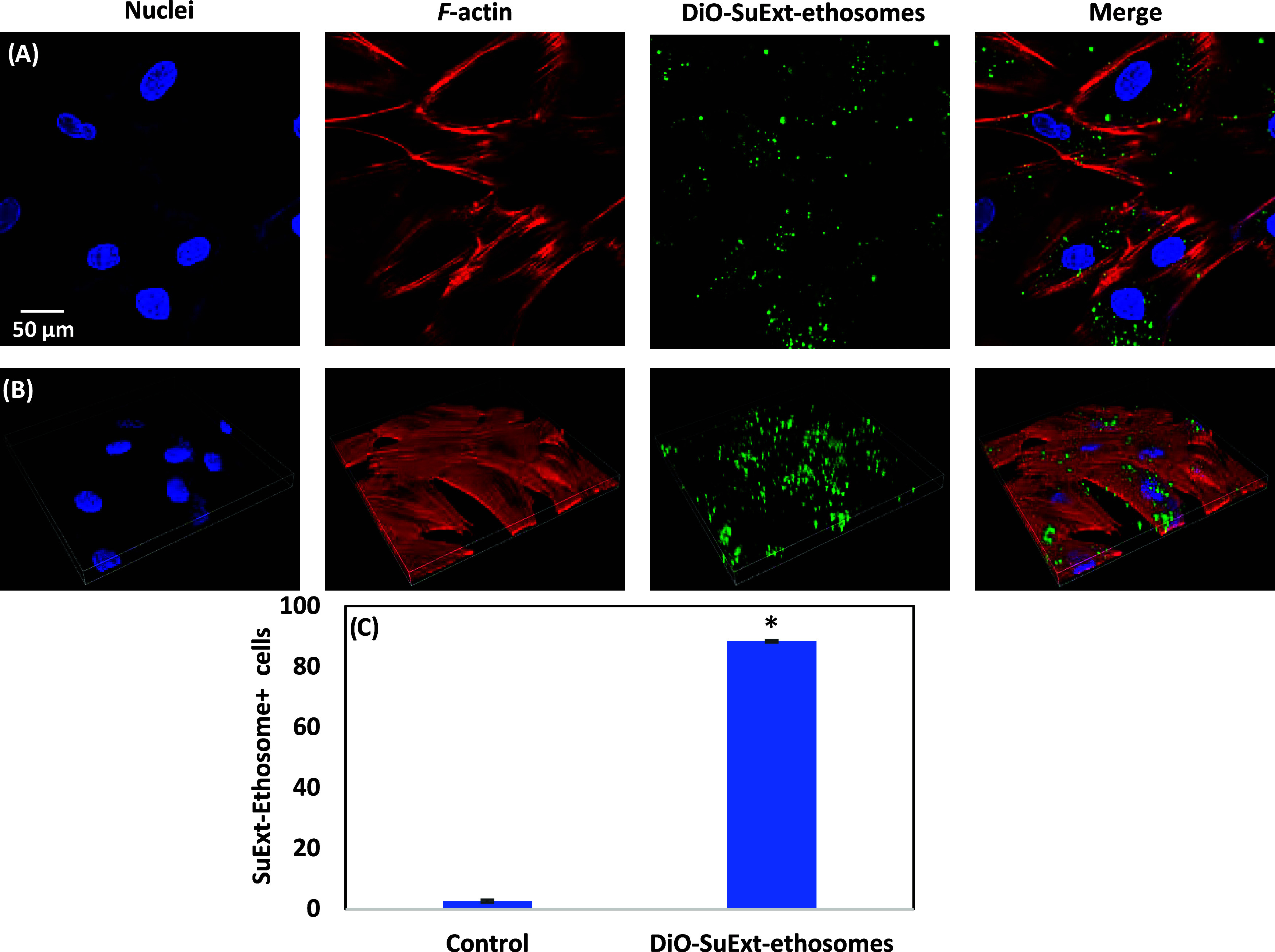
Cellular uptake investigation. Representative confocal images of HDFs treated with DiO-SuExt-ethosomes (100 μg/mL) for 24 h (DiO-SuExt-ethosomes in green, f-actin in red, and nuclei in blue): single z-stack (A) and 3D rendering (B) images. (C) Quantitative evaluation of the uptake through flow cytometry (*n* = 3, *p < 0.05).

The high internalization extent of the ethosomes has been attributed to the ethanol presence in their structure, which intercalates with the polar headgroup of the cell membrane, thus enhancing membrane permeability and facilitating nanoparticle fusion with the cellular membrane.^38^ The observed uptake confirms that the ethosomal nanoparticles represent an efficient delivery system into cells, despite the fact that we cannot perform a direct comparison with the internalization of the “free” nonencapsulated SuExt. Entrapment into ethosomes was pursued based on the hypothesis that this vesicular carrier would improve not only stability and skin permeation but potentially cellular internalization as well. In this regard, a comparative study evaluated the internalization of ethosomes and liposomes by human embryonic skin fibroblast cells (CCC-ESF-1): a 2-fold increment of ethosome uptake with respect to liposomes was found, already after 4 h of incubation.^39^

### 3.3 Potentiality of SuExt-Ethosomes in Wound Healing

Fibroblasts and keratinocytes at the edge of the wound area contribute to its closure by proliferating or migrating to restore the skin barrier, protecting against microbial infection and toxicant exposure. Skin fibroblasts are likely to proliferate until contact inhibition occurs and to move toward the center of the injured region, covering the wound area. The wound closure capacity of the cells is commonly assessed through a scratch assay, which involves the observation of the closure of an artificial gap created in a cell monolayer.

In this study, HDF cultures have been exploited as a model to check wound closing upon different treatments (SuExt, ethosomes, and SuExt-ethosomes). Representative images of the experiment are reported in Figure 4A, whereas a quantitative evaluation is reported in Figure 4B. Gaps at time zero are normalized to 100% of the wound area. After 24 h, the wound area was significantly reduced just in the SuExt (58.0 ± 3.9%) and SuExt-ethosome (50.5 ± 3.1%) experimental groups. Statistical analysis of the wound healing assay revealed no significant difference between the SuExt and SuExt-ethosome treatment groups, suggesting that ethosomal encapsulation did not markedly enhance the biological activity of the extract under the conditions tested. While both formulations significantly improved wound closure compared with the control, the lack of difference between SuExt and its ethosomal form indicates that the extract may already be efficiently bioavailable and active in its free form. However, the potential benefits of ethosomal encapsulation—such as improved physicochemical stability, prolonged release, enhanced permeation *in vivo*, and protection of sensitive phytochemicals—may not be fully reflected in this short-term *in vitro* wound healing model.

**Figure 4:**
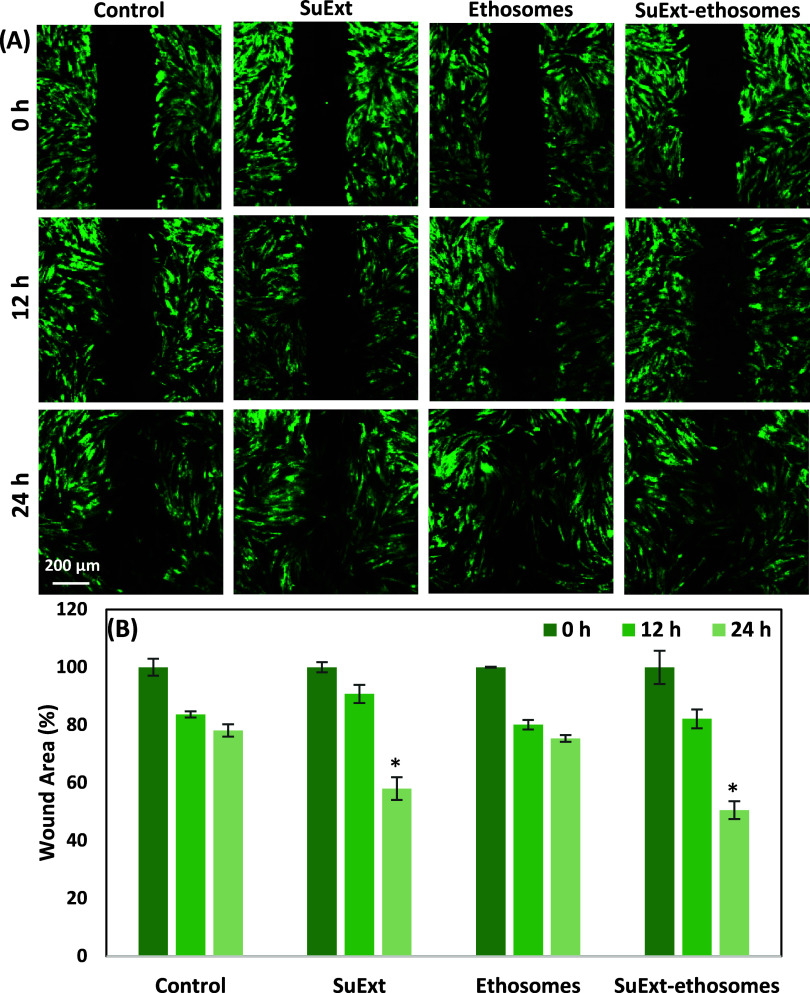
*In vitro* scratch assay on HDF cultures after different treatments: representative fluorescence images (A) and quantitative evaluation (B). Data are presented as mean value ± standard deviation (*n* = 3, *p < 0.05).

Obtained findings, although preliminary, are in line with other literature data: as an example, *Polygonum cuspidatum* extract was reported to promote wound closure by modulating TGF-1 expression, leading to accelerated myofibroblast transformation through an enhanced collagen expression.^40^ This comparison is relevant as both *Rhus coriaria* and *Polygonum cuspidatum* are known to be rich in phenolic compounds, particularly resveratrol derivatives and flavonoids, which have been implicated in modulating cellular migration and wound repair pathways through antioxidant and anti-inflammatory mechanisms. Although the botanical sources differ, the alignment of bioactive profiles and biological responses supports the hypothesis that similar phytoconstituents may underlie comparable cellular outcomes.

The antioxidant activity of SuExt-ethosomes was assessed on HDFs subjected to oxidative stress, induced by treating the cells with 200 μM *tert*-butyl hydroperoxide (tBH). Following oxidative stress induction, the cells were treated with SuExt, ethosomes, and SuExt-ethosomes. Results (Figures 5A and S3A) show strong oxidative stress production in insulted cultures (22.0 ± 0.8% of ROS^+^ cells); however, a significant reduction was observed in cultures treated with SuExt (15.1 ± 2.4%) and SuExt-ethosomes (12.4 ± 1.1%). No statistically significant difference was observed between the SuExt and SuExt-ethosome treatment groups; interestingly, plain ethosomes also exhibited antioxidant activity, suggesting that the phospholipid components of the carrier system may contribute to the overall redox modulation. These findings point to a potential synergistic or additive effect between SuExt and the ethosomal formulation, and this indeed translates into an increased antioxidant activity of SuExt-ethosomes in reducing oxidative stress in HDFs.

**Figure 5:**
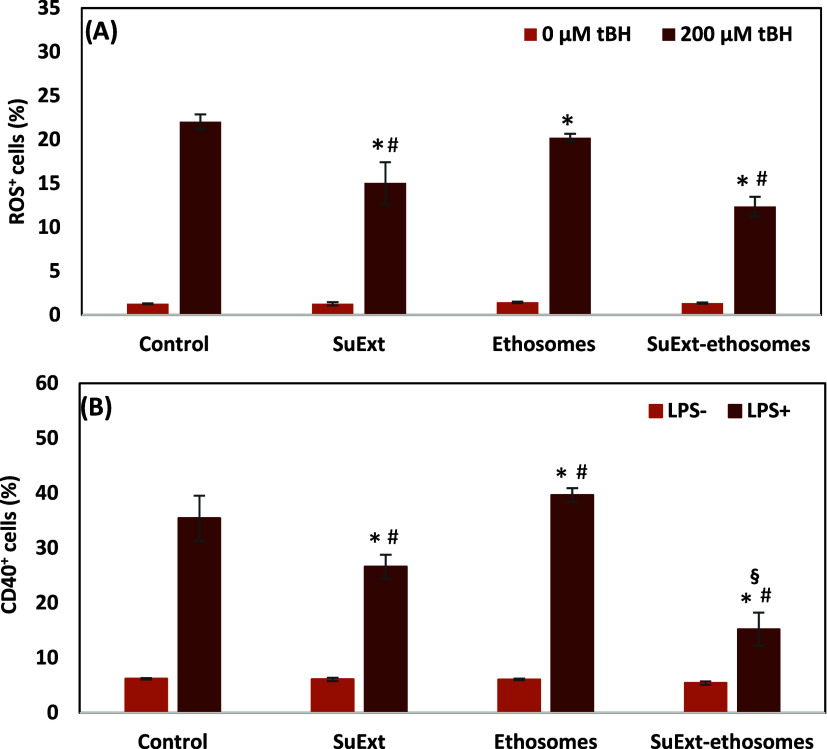
ROS evaluation in insulted (tBH^+^) and noninsulted (tBH^–^) cultures (A) and CD40 expression evaluation in insulted (LPS^+^) and noninsulted (LPS^–^) cultures (B) upon different treatments. Data are presented as mean value ± standard deviation (*n* = 3, *p < 0.05 with respect to control, ^#^ *p* < 0.05 with respect to ethosomes, ^§^ *p* < 0.05 with respect to SuExt).

Literature already provides evidence of the antioxidant activity of sumac extract, for example, in HMEC-1 endothelial cells. Cultures were exposed to UV-A radiation (20 J/cm^2^) and then analyzed for ROS levels: sumac-treated cells showed significantly lower oxidant levels, and this reduction was attributed to the activation of the oxidative defense mediator Nrf-2.^41,42^

SuExt contains a wide variety of organic acids, particularly glycolic acid, citric acid, lactic acid, malic acid, and gallic acid, all of which possess strong antioxidant activity.^18^ Betaine, the most abundant compound in the extract, plays a crucial role in the synthesis of nonenzymatic antioxidants such as glutathione.^43^ Choline, the third most representative compound in the extract, contributes to immune system regulation by inhibiting TNF-α, IL-1, and TGF-β expression.^12^ Additionally, trigonelline aids in suppressing the unfolded protein-dependent oxidative response while stimulating antioxidant enzyme activities.^44^ Beyond these key components, many other biologically active molecules, including amino acids, vitamins, and metabolites, contribute to antioxidant properties through different mechanisms. The higher antioxidant activity of SuExt-ethosomes with respect to SuExt alone can be attributed to the combined oxidant-reducing effects of ethosomes; furthermore, this can be attributed to the protective effects of the ethosomes on the antioxidant activity of their cargo. This phenomenon is supported by a study showing that the encapsulation of the *Ginkgo biloba* extract in phosphatidylcholine-based liposomes significantly increases the antioxidant activity of the extract.^45^ The relevance of this comparison lies in the presence of overlapping classes of antioxidant phytochemicals, such as flavonoids and terpenoids, which act through shared molecular mechanisms. The cellular response patterns suggest that plant-derived antioxidants may exert convergent biological effects, particularly in oxidative-stress-associated dermal models.

Inflammatory responses play a crucial role in creating a protective barrier against environmental toxicants and microorganisms during the wound healing process.^46,47^ Initially, the CD40 presentation by skin fibroblast cells induces the release of cytokines such as IL-1ß, IL-6, and TNF-α, which stimulate the inflammatory pathway.^48^ However, prolonged CD40 presentation can lead to excessive cytokine secretion, which may result in redundant fibrosis production and eventual scar tissue formation. Additionally, excessive concentrations of cytokines can trigger abnormal inflammatory responses in keratinocytes and endothelial cells within the skin.^49^ Therefore, regulating CD40 expression in fibroblasts is considered a key approach to modulating the inflammatory response.^50,51^

To assess the effects on CD40 presentation, HDFs were first exposed to lipopolysaccharide (LPS), a known CD40 stimulant. The cultures were then treated with SuExt, ethosomes, and SuExt-ethosomes. Subsequently, the cultures were immunostained for CD40 and analyzed by flow cytometry. The results (Figures 5B and S3B) show a significant reduction in CD40 expression (26.6 ± 2.2 and 15.2 ± 3.0% of CD40^+^ cells treated with SuExt and SuExt-ethosomes, respectively) with respect to the control group (35.4 ± 4.1%) and to the cultures treated with plain ethosomes (39.7 ± 1.2%). The treatment with SuExt-ethosomes resulted in a significantly reduced CD40 expression with respect to the treatment with plain SuExt: this result can be attributed to the synergistic or additive effect between SuExt and the ethosomal formulation or to an enhanced uptake of extract mediated by the nanoparticles.

Previous studies have shown that flavonoids, a subgroup of phenolics, exert an anti-inflammatory effect by modulating the CD40 expression. In particular, luteolin has been identified as a potent downregulator of CD40, along with lovastatin, which inhibits the NF-κB activation in human vascular endothelial cells.^52,53^ Additionally, tanshinone IIA has been reported to downregulate CD40 expression in human umbilical vein endothelial cells (HUVECs), particularly when these cells are stimulated through H_2_O_2_.^54^ The abundance of flavonoids present in SuExt, loaded into ethosomes, thus likely contributes to the observed reduction of the CD40 expression in HDFs.

Proteomics was eventually performed to elucidate the molecular pathways affected by the treatments (Figure 6). In all cases, we found a statistically significant variation in the protein expression profile of the HDFs; specifically, the comparison was made between SuExt-ethosomes-treated and control cells (Figure 6A, differentially expressed proteins in Table S3), SuExt-ethosomes- and ethosomes-treated cells (Figure 6B, differentially expressed proteins in Table S4), and SuExt-ethosomes- and SuExt-treated cells (Figure 6C, and differentially expressed proteins in Table S5).

**Figure 6:**
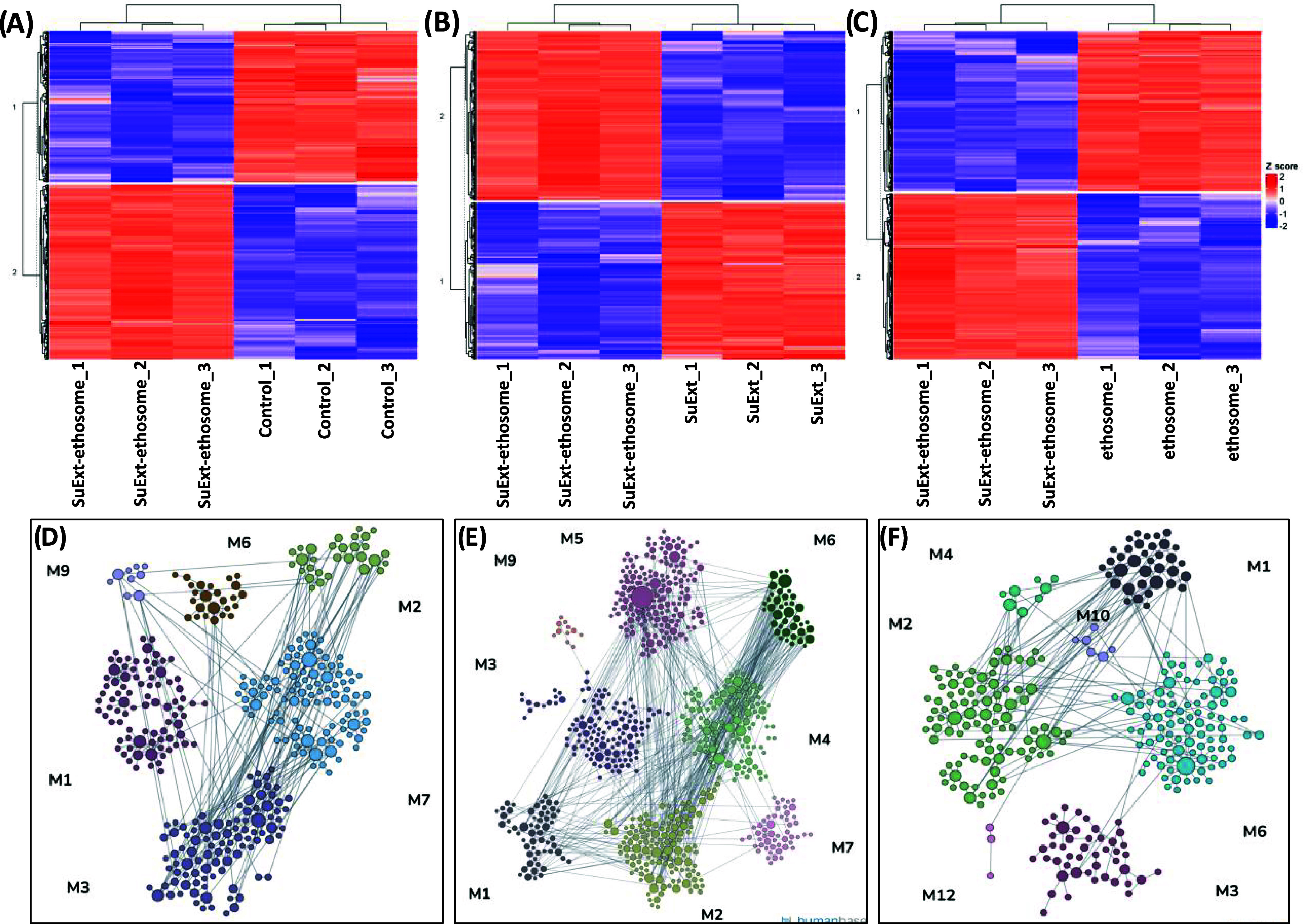
Proteomics. Heatmaps showing the protein differential expression for (A) control vs SuExt-ethosomes-treated cells and (B) ethosomes-treated cells vs SuExt-ethosomes-treated cells. (C) SuExt-treated cells vs SuExt-ethosomes-treated cells. GO enrichment for biological function: node map relative to (D) SuExt-ethosomes-treated cells vs control cells, (E) SuExt-ethosomes-treated cells vs ethosomes-treated cells, and (F) SuExt-ethosomes-treated cells vs SuExt-treated cells. Red, overexpressed protein; blue, underexpressed proteins; and the values are normalized using Z-score. The single proteins present in each cluster are listed in Tables S3–S5.

The comparison between SuExt-ethosome-treated cells and control cells revealed the enrichment of GO terms such as cellular response to decreased oxygen levels (Figure 6D and Table S3, group M1), response to hydrogen peroxide (Figure 6D and Table S3, group M2), positive regulation of protein polymerization (Figure 6D and Table S3, group M7), and mitotic spindle organization (Figure 6D and Table S3, group M9). These findings highlight the effects of SuExt-ethosomes on cellular responses to oxygen reduction and hydrogen peroxide exposure, as well as their positive impact on cell proliferation. The overexpression of sirtuin 2 (SIRT2) in SuExt-ethosomes-treated cells is a hint of altered antioxidant and redox-mediated cellular homeostasis. Notably, one of the targets of SIRT2, PGC-1α, is deacetylated by SIRT2, which, in turn, modulates mitochondrial biogenesis. This process has been associated with the upregulation of antioxidant enzyme expression and a reduction in ROS levels.^55^

SuExt-ethosome treatment led to the downregulation of APOA1, an apolipoprotein that regulates cell-to-cell junctions by inducing α-actin depolymerization.^56^ While cell-to-cell junctions among fibroblasts are essential for maintaining the barrier structure of the dermis and epidermis, these junctions can hinder wound healing by restricting cell motility across decellularized skin areas. The downregulation of APOA1 promotes cell migration by facilitating the movement of fibroblasts into the wound. Cell migration also relies on the coordinated reorganization of the cytoskeleton, including lamellipodial extension, formation of forward adhesions, and exertion of a contractile force to pull the cell body forward. Profilin 2 (PFN2), which upregulates actin filament polymerization, was upregulated in SuExt-ethosome-treated cells. This upregulation is likely responsible for enhanced migration, as α-actinins are known to interact with several proteins that regulate both actin and cellular motility.^57^ PTPRJ, a tyrosine phosphatase receptor, also regulates various biological responses, such as cell proliferation, migration, and cellular communication, and promotes stronger linkage among adherent junction components.^58^ Furthermore, SuExt-ethosome treatment downregulated the echinoderm microtubule-associated protein-like 2 (EML2), which inhibits cell motility.^59^ Altogether, these changes in protein expression induced by SuExt-ethosomes help explain the enhanced cell migration observed in the scratch assay.

RFC4 is a replication factor that plays a crucial role in cellular survival against UV-induced DNA damage by participating in endoduplication.^60^ The overexpression of RFC4 in SuExt-ethosomes-treated cells suggests the activation of a DNA damage-repairing mechanism, thus reducing genetic code alterations. UHRF1, a ubiquitin E3 ligase, is an epigenetic regulator that coordinates DNA methylation and histone modifications. Additionally, its role in DNA damage repair has been explored and recognized as crucial for maintaining the epigenome, where it also functions as a senescence suppressor. Considering this, the upregulation of UHRF1 in SuExt-ethosomes-treated cells suppresses cell senescence by maintaining epigenomic integrity.^61^ Daxx is induced upon exposure to UV irradiation and hydrogen peroxide via impaired MKK/c-Jun-N-terminal kinase (JNK) activation. Its overexpression in SuExt-ethosomes-treated cells suggests an altered sensitivity to oxidative stress-dependent damage, potentially enhancing the programmed cell death response.^62^ Nicotinamide adenine dinucleotide (NAD)-dependent tubulin deacetylase (SIRT2) suppresses senescence by inducing cell cycle arrest and increasing the susceptibility of damaged cells to apoptosis. The upregulation of this protein in SuExt-ethosomes-treated cells suggests improved sensitivity to damaged cells, thereby inhibiting senescence.^63^ Overall, SuExt-ethosomes may play a crucial role in either repairing or eliminating damaged cells, particularly in response to oxidative stress exposure.

COL1A1, collagen type I A1, is regulated by the Wnt/β-catenin signaling activity targeted by cyclin D1 and, as a result, downregulated in SuExt-ethosomes-treated cells, implying an effect on the G1/S phase transition during the cell cycle.^64^ Moreover, overexpression of the heat shock protein 90AA1 (HSP90AA1) is in line with the downregulated expression of COL1A1, indicating the potentiality in wound healing of SuExt-ethosomes.^65^

The comparison between SuExt-ethosomes-treated cells and ethosomes-treated cells revealed statistically significant GO term enrichments, including telomere maintenance via telomerase (Figure 6E and Table S4, group M2), cellular response to oxygen levels (Figure 6E and Table S4, group M3), response to increased oxygen levels (Figure 6E and Table S4, group M5), and mitochondrial membrane organization (Figure 6E and Table S4, group M6). The first GO term enrichment indicates the potential positive effects of SuExt-ethosomes on wound healing by supporting the adequate proliferation of fibroblasts, keratinocytes, and endothelial cells. This proliferation is essential for tissue regeneration, extracellular matrix deposition, and new blood vessel formation. Telomere maintenance in these cells is mediated by chaperone TCP-1 ring complex (TRiC) overexpression, which has been recently identified as a mediator of telomerase recruitment and localization, as well as a factor that contributes to the addition of telomeric repeats to chromosome ends.^66^ In view of this, telomer-shortening-dependent cellular aging is diminished in these cells.

The observed enrichment of the response to increased oxygen levels GO term is strictly related to SuExt and suggests that alterations in response to oxygen levels improve critical aspects of wound healing by supporting key physiological processes. In particular, these processes include the production of ATP, which is crucial for cellular activities such as proliferation and migration. Focusing on the effects induced by SuExt-ethosomes, IFIH1, a helicase C domain 1 protein, plays a role in stimulating an inflammatory response by promoting IFN-β and TNF-α cytokine secretion. The downregulation of IFIH1 in SuExt-ethosomes-treated cells suggests the anti-inflammatory effect of SuExt, which is consistent with the previous data.^67^ The downregulatory effect on IFIH1 and CD40 expressions may be involved in the reduction of excessive cytokine secretion; furthermore, the presence of compounds enhances cellular oxidative stress defenses, supporting fibroblast migration and proliferation, which are fundamental for effective wound closure.

MAPK1 (mitogen-activated protein kinase 1) is a key mediator of serine/threonine kinase activation, regulating telomerase activity, matrix recognition, migration, and fibronectin organization. Integrin-linked serine/threonine kinases, which make up a large family of heterodimeric extracellular matrix (ECM) receptors, bind to ECM proteins such as fibronectin and collagen to mediate cellular adhesion and migration. SuExt-ethosomes induce MAPK1 overexpression that may trigger the adhesion and migration response of HDFs in the presence of external stimuli.^68^ Additionally, casein kinase II (CSNK2A1) is overexpressed and plays a similar role in HDFs.^69^

SuExt-ethosomes promote the overexpression of STAT3, a critical transcription factor that plays a crucial role in wound closure by promoting the expression of collagen and other extracellular matrix components, such as matrix metalloproteinases (MMPs) in dermal fibroblasts. The process of tissue formation can be arbitrarily divided into three phases (re-epithelialization, formation of granulation tissue, and neovascularization), and it is observed that STAT3 is overexpressed in these events.^53^ Additionally, the ubiquitin-specific protease 7 (USP7) protein is overexpressed in the presence of SuExt-ethosomes, contributing to wound closure by stimulating the expression of the TGF-β1 protein.^70^ The phosphatase and tensin homologue protein (PTEN) helps in regulating tissue repair by dephosphorylating tissue repair proteins when they are excessively expressed, thereby preventing extreme fibrotic activity and scar tissue formation. SuExt-ethosomes induce PTEN overexpression that may effectively manage the wound repair process and prevent the development of excessive scarring.^71^

The comparison between SuExt-ethosomes- and SuExt-treated cells revealed statistically significant GO term enrichments, including regulation of cell cycle arrest (Figure 6F and Table S5, group M3) and signal transduction involved in mitotic DNA damage checkpoint (Figure 6F and Table S5, group M4). These results again highlight the role of ethosomes in regulating cell proliferation control mechanisms. The protein expression profiles of the SuExt-ethosomes- and SuExt-treated HDFs were comparatively evaluated to determine the effects of ethosomes on the cells. An upregulation of HSP90AA1 (a heat shock protein) was found both in the SuExt-ethosome group vs the SuExt group and in the SuExt-ethosome group vs control, confirming its ethosome-mediated overexpression. This result can be attributed to the phosphatidylcholine-induced cell metabolic activity, which is particularly significant in the context of wound healing. Stathmin 1 (STMN1) is a mitotic regulator and plays an important role in the maintenance of cell biological characteristics by regulating rapid microtubule remodeling in cell proliferation.^72^ The downregulation of the STMN1 expression in ethosomes-treated cells suggests suppressed proliferation, also proven by the TPM1 cell proliferation suppressor marker overexpression.^73^ CDK4, a cyclin-dependent kinase 4 protein, is involved in many cellular processes, including cell proliferation and survival. The downregulation of CDK4 in response to ethosome treatment further confirms the proliferation inhibitory activity of these nanoparticles.^74^ Moreover, the overexpression of ACADVL (long-chain acyl coenzyme A dehydrogenase) plays an important role in long-chain fatty acid oxidation and can be explained by the ethosomal phosphatidylcholine accumulation and metabolism in the cells.^75^ Overall, the protein expression profile in the presence of ethosomes highlights an effect on the proliferation activity, which could serve as a regulatory mechanism to prevent uncontrolled cell proliferation during wound healing.

The release mechanism of SuExt from SuExt-ethosomes following cellular uptake plays a pivotal role in modulating the just-described biomolecular pathways. Ethosomes, due to their ethanol-rich composition, enhance membrane permeability and facilitate nanoparticle fusion with cellular membranes, enabling efficient internalization. Once inside the cells, the release of the extract is influenced by intracellular conditions, such as enzymatic degradation of the ethosomal bilayers and endosomal escape, ensuring a controlled and sustained delivery of bioactive compounds. This release impacts key regulatory pathways involved in wound healing.

## 4 Conclusion

SuExt contains a wide variety of active compounds, many of which exhibit potent antioxidant and anti-inflammatory properties. To enhance their potential in nanomedicine, ethosome nanocarriers, known for their high intra- and transdermal penetration capabilities, were exploited for encapsulation, particularly for addressing skin-related issues. Our findings demonstrate the successful formulation of SuExt-ethosomes, showcasing their favorable stability, cytocompatibility, and effective ROS scavenging activity, along with CD40-dependent inflammation inhibition in HDFs. Overall, these results offer valuable insights into the benefits of SuExt-ethosomes and strongly support further *in vivo* studies to fully exploit their therapeutic potential; in particular, further studies involving skin permeation assays and long-term biological evaluations would help determine whether the formulation indeed offers advantages beyond immediate wound healing.

## Supplementary Material



## Data Availability

Proteomics data are available via ProteomeXchange with identifier PXD059559. All other data are available from the Corresponding Authors upon reasonable request [please send email to gianni.ciofani@iit.it].

## Notes

HPLC plot and calibration curve; internalization investigation representative flow cytometry plots; ROS levels and CD40 expression representative flow cytometry plots; semiquantitative extract characterization; and enriched GO terms for the different comparisons (PDF)
