# Supplementary material for: Evaluation of the Effects of Sumac (Rhus coriaria) Extract-Loaded Ethosomes on an In Vitro Wound Healing Model
Source: ACS Omega. 2025 Jun 18;10(25):26669–83. doi: 10.1021/acsomega.5c00910 (PMC12224110; doi:10.1021/acsomega.5c00910)
Supplement: Supplementary file 1 [file ao5c00910_si_001.pdf]

Supplementary Information

Evaluation of the Effects of Sumac (*Rhus coriaria*) Extract-Loaded Ethosomes on an *in Vitro* Wound Healing Model

Melis Emanet[1,], Matteo Battaglini[1], Alessio Carmignani[1], Federico Catalano[2], Martina Bartolucci[3], Andrea Petretto[3], Gianni Ciofani[1,*]*

[1]Istituto Italiano di Tecnologia, Smart Bio-Interfaces, Viale Rinaldo Piaggio 34, 56025 Pontedera, Italy

[2]Istituto Italiano di Tecnologia, Electron Microscopy Facility, Via Morego 30, 16163 Genova, Italy

[3]IRCCS Istituto Giannina Gaslini, Clinical Proteomics and Metabolomics Core Facility, Via Gerolamo Gaslini 5, 16147 Genova, Italy

*Corresponding Authors: melis.emanetciofani@iit.it, gianni.ciofani@iit.it

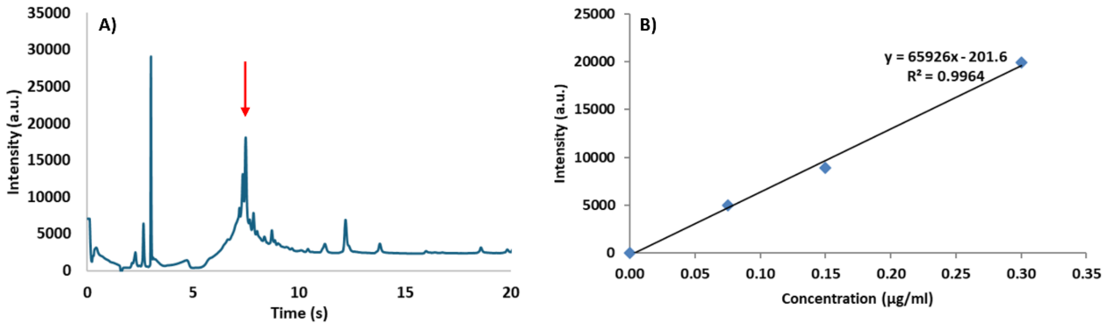

Figure S1. Representative HPLC spectrum (A), with the indication of the peak selected for further evaluation (red arrow), and standard curve (B) for SuExt concentration quantification.

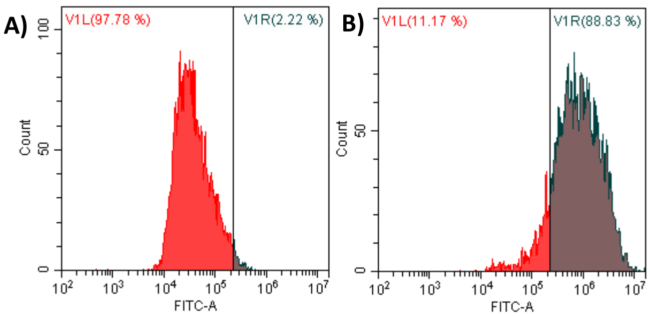

Figure S2. Cellular up-take investigation. Representative flow cytometry fluorescence plot of control (A) and DiO-SuExt-ethosomes-treated (B) HDF cultures.

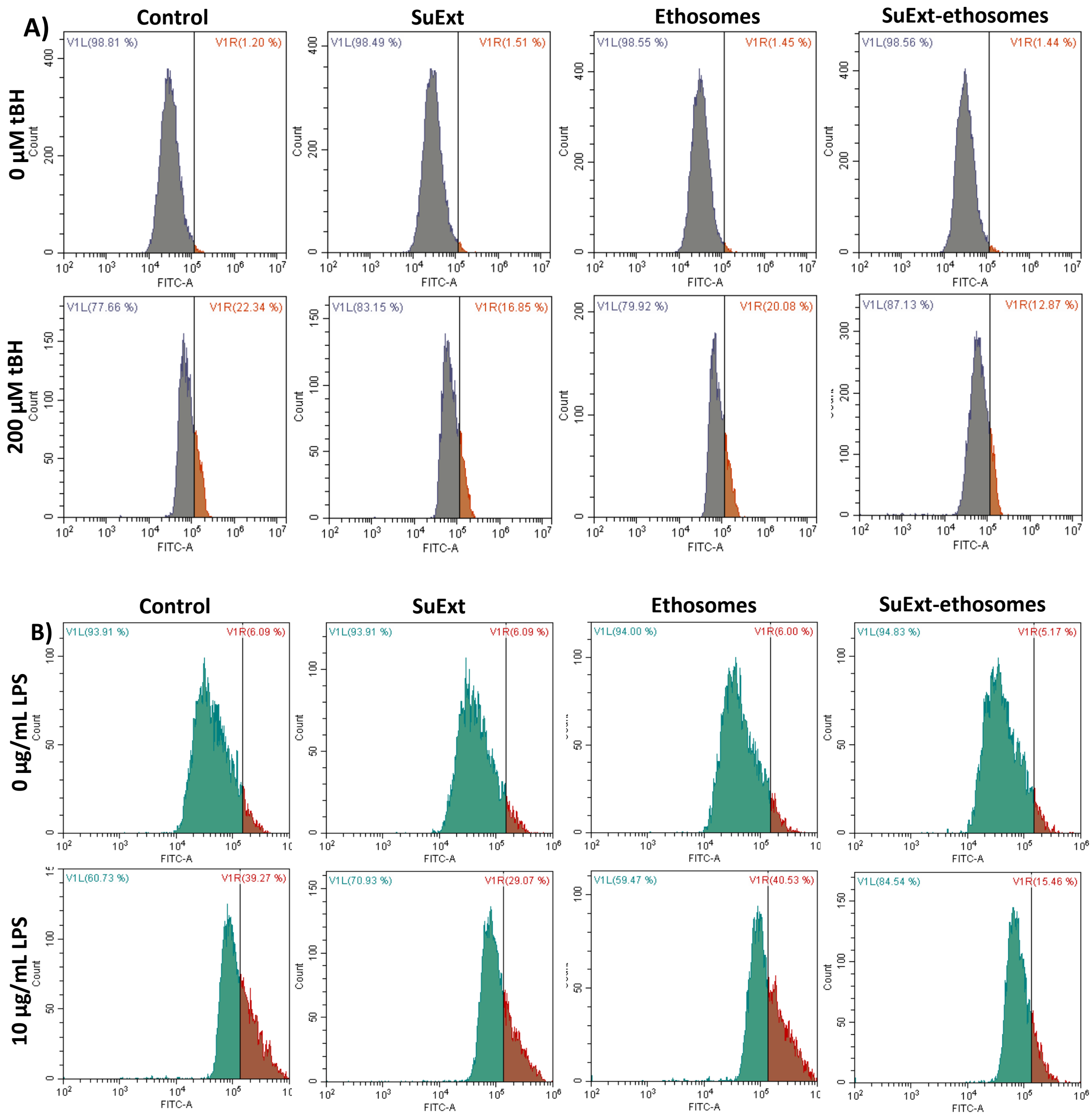

Figure S3. Representative flow cytometry fluorescence plot of (A) ROS levels in oxidative stress-induced (tBH+) or not-induced (tBH-) HDFs after 24 h of the indicated treatments and (B) of CD40 expression levels in inflammation-induced (LPS+) or not-induced (LPS-) HDFs after 24 h of the indicated treatments.

Table S1. Sumac extract characterization: metabolites identified in the negative polarity runs.

% Area	Compound
32,73	DL-Malic acid
30,79	Citric acid
29,55	Gallic acid
29,50	D-(+)-Malic acid
27,83	Xylenesulfonate
27,56	1,2,3,4-Tetrakis-O-(3,4,5-trihydroxybenzoyl)-?-D-glucopyranose
27,01	Methylmalonic acid
26,97	Myricitrin
26,87	Neochlorogenic acid
26,56	Gentisic acid
26,31	Afzelin
26,23	5-(5,7-Dihydroxy-3-methoxy-4-oxo-4H-chromen-2-yl)-2-hydroxyphenyl beta-D-xylopyranoside
25,98	18-?-Glycyrrhetinic acid
25,72	Chlorogenic acid
25,54	Salicylic acid
25,41	D-(+)-Galactose
25,02	Quercetin
24,88	(2R)-2,3-Dihydroxypropanoic acid
24,53	NP-021050
24,50	NP-000921
24,29	16-Hydroxyhexadecanoic acid
23,86	3,4,5-trihydroxycyclohex-1-ene-1-carboxylic acid
23,83	Luteolin
23,26	myricetin 3-O-beta-D-galactopyranoside
23,03	Corchorifatty acid F
22,94	(+/-)12(13)-DiHOME
22,53	Rutin
22,50	NP-014113
22,03	Apigetrin
21,92	(+/-)9,10-dihydroxy-12Z-octadecenoic acid

Table S2. Sumac extract characterization: metabolites identified in the positive polarity runs.

% Area	Compound
30,63	Betaine
29,01	Choline
28,29	Proline
27,29	Trigonelline
26,25	L-Phenylalanine
26,41	D-(+)-Glucose
26,04	Myricitrin
25,56	Pipecolic acid
25,20	3-Hydroxy-3-methylbutanoic acid
25,88	(1r,3R,4s,5S)-4-{[(2E)-3-(3,4-dihydroxyphenyl)prop-2-enoyl]oxy}-1,3,5-trihydroxycyclohexane-1-carboxylic acid
25,52	2-(3,4-dihydroxyphenyl)-5,7-dihydroxy-3-{[(2S,3R,4R,5R,6S)-3,4,5-trihydroxy-6-methyloxan-2-yl]oxy}-4H-chromen-4-one
26,07	D-(+)-Pyroglutamic Acid
25,12	DL-Arginine
25,22	Agmatine
24,88	?-Lactose
24,78	L-Glutamic acid
24,65	(2S,3R,4S,5R,6R)-3,4,5-tris(3,4,5-trihydroxybenzoyloxy)-6-[(3,4,5-trihydroxybenzoyloxy)methyl]oxan-2-yl 3,4,5-trihydroxybenzoate
24,39	N-Phenyl-5-(4-(trifluoromethyl)phenyl)oxazol-2-amine
24,46	7-{[(2S,3R,4S,5S,6R)-4,5-dihydroxy-6-(hydroxymethyl)-3-{[(2S,3R,4R,5R,6S)-3,4,5-trihydroxy-6-methyloxan-2-yl]oxy}oxan-2-yl]oxy}-5-hydroxy-2-(4-hydroxyphenyl)-3-{[(2S,3R,4S,5S,6R)-3,4,5-trihydroxy-6-({[(2R,3R,4R,5R,6S)-3,4,5-trihydroxy-6-methyloxan-2-yl]oxy}methyl)oxan-2-yl]oxy}-4H-chromen-4-one
24,36	NP-019748
24,92	D-(+)-Maltose
24,27	Myricetin
24,18	NP-008882
24,19	Oleamide

24,14	(2E)-3-(4-{[(2S,3R,4S,5S,6R)-3,4,5-trihydroxy-6-(hydroxymethyl)oxan-2-yl]oxy}phenyl)prop-2-enoic acid
24,16	Asparagine
24,14	Kaempferol
25,75	4-Guanidinobutyric acid
23,96	NP-013663
23,94	Maltol
23,56	L-Pyroglutamic acid
23,71	(?)13-HpODE
25,10	Prolylleucine
23,67	D-Raffinose
23,60	(2S,3R,4S,5R,6R)-5-hydroxy-6-(hydroxymethyl)-3,4-bis(3,4,5-trihydroxybenzoyloxy)oxan-2-yl 3,4,5-trihydroxybenzoate
23,45	(2S,3R,4S,5S,6R)-4,5-dihydroxy-2-(3,4,5-trihydroxybenzoyloxy)-6-[(3,4,5-trihydroxybenzoyloxy)methyl]oxan-3-yl 3,4,5-trihydroxybenzoate
23,45	3-Hydroxypicolinic acid
26,49	L-Isoleucine
23,23	D-(-)-Aspartic acid
23,43	L-Tyrosine
23,46	2,3,4,9-Tetrahydro-1H-?-carboline-3-carboxylic acid
23,45	JWH-198
28,07	Adenine
23,35	(2S,3R,4R,5S,6S)-2-{[2-(3,4-dihydroxyphenyl)-5,7-dihydroxy-4-oxo-4H-chromen-3-yl]oxy}-3,5-dihydroxy-6-methyloxan-4-yl 3,4,5-trihydroxybenzoate
23,16	4-(4-chlorophenyl)-2-(3-pyridyl)-1,3-thiazole hydrobromide
24,22	9-Oxo-10(E),12(E)-octadecadienoic acid
25,74	Afzelin
23,06	(2R,3R,4R,5R,6S)-2-{[(2R,3S,4S,5R,6R)-6-(benzyloxy)-3,4,5-trihydroxyoxan-2-yl]methoxy}-6-methyloxane-3,4,5-triol
23,05	Schaftoside
23,03	myricetin 3-O-beta-D-galactopyranoside
22,99	5,5',7,7'-tetrahydroxy-2,2'-bis(4-hydroxyphenyl)-4H,4'H-[8,8'-bichromene]-4,4'-dione
22,95	Oleoyl ethanolamide

22,70	3,5-dihydroxy-2-(4-hydroxyphenyl)-6-(3-methylbut-2-en-1-yl)-7-{[3,4,5-trihydroxy-6-(hydroxymethyl)oxan-2-yl]oxy}-3,4-dihydro-2H-1-benzopyran-4-one
25,26	Guanine
22,68	(2S,3S,4S,5R,6R)-6-[3-(benzoyloxy)-2-hydroxypropoxy]-3,4,5-trihydroxyoxane-2-carboxylic acid
25,59	2-(3,4-Dihydroxyphenyl)-5,7-dihydroxy-4-oxo-4H-chromen-3-yl 6-O-?-D-xylopyranosyl-?-D-glucopyranoside
22,72	Nicotinamide
22,72	L-Iditol
23,23	(4S)-4-hydroxy-3,5,5-trimethyl-4-[(1E)-3-{[(2R,3R,4S,5S,6R)-3,4,5-trihydroxy-6-(hydroxymethyl)oxan-2-yl]oxy}but-1-en-1-yl]cyclohex-2-en-1-one
22,66	Rutin
22,52	?-Linolenic acid
22,56	NP-000587
22,45	Apigetrin
24,67	Linoleoyl ethanolamide
22,31	Piperine
22,37	Citroflex A-4
22,99	1-Linoleoyl glycerol
23,76	Nicotinic acid
23,75	Quercetin
21,90	Adenosine
22,02	NP-020921
21,94	Palmitoyl ethanolamide
21,95	DL-Tryptophan
21,92	Corymboside
21,91	trans-3-Indoleacrylic acid
21,86	2-(3,4-Dihydroxyphenyl)-5-hydroxy-4-oxo-4H-chromen-7-yl 6-O-(6-deoxy-alpha-L-mannopyranosyl)-beta-D-glucopyranoside
21,84	NP-011548

Table S3. List of the enriched GO terms for biological functions relative to SuExt-ethosomes-treated cells *vs.* control cells.

MODULE	TOP TERMS (Max 10)	Q VAL	GENES	TERMS
M1	positive regulation of epithelial to mesenchymal transition	0.00067635	84	130
	regulation of cell adhesion molecule production	0.00243672		
	regulation of epithelial to mesenchymal transition	0.00243672		
	cell adhesion molecule production	0.00391440		
	negative regulation of heterotypic cell-cell adhesion	0.00434160		
	Cdc42 protein signal transduction	0.00609669		
	regulation of cell-cell adhesion involved in gastrulation	0.00609669		
	regulation of endothelial cell migration	0.00628614		
	epithelial to mesenchymal transition	0.00667014		
	collagen metabolic process	0.00667014		
M2	negative regulation of receptor-mediated endocytosis	0.00845471	94	38
	negative regulation of lipid transport	0.01103509		
	negative regulation of type I interferon production	0.01103509		
	positive regulation of stress fiber assembly	0.01547946		
	positive regulation of actin filament bundle assembly	0.02072409		
	negative regulation of lipid localization	0.02150774		
	negative regulation of endocytosis	0.02150774		
	regulation of stress fiber assembly	0.02565877		
	negative regulation of response to biotic stimulus	0.02703154		
	cellular iron ion homeostasis	0.02830482		
M3	regulation of lipid transport by regulation of transcription from RNA polymerase II promoter	0.00243672	107	91
	histone H2A acetylation	0.00696106		
	cellular response to X-ray	0.00817828		
	phosphatidylinositol-3-phosphate biosynthetic process	0.00901959		
	histone H4 acetylation	0.00901959		
	regulation of telomere capping	0.01091039		
	positive regulation of response to DNA damage stimulus	0.01105395		
	regulation of DNA-templated transcription in response to stress	0.01105395		
	activation of MAPKK activity	0.01275731		
	positive regulation of response to endoplasmic reticulum stress	0.01275731		
M4	cell cycle arrest	0.00609669	53	27
	negative regulation of cell cycle arrest	0.00609669		
	positive regulation of release of cytochrome c from mitochondria	0.00696106		
	positive regulation of cysteine-type endopeptidase activity	0.00845471		
	regulation of release of cytochrome c from mitochondria	0.00901959		
	positive regulation of protein complex disassembly	0.00901959		
	regulation of cell cycle arrest	0.01040861		
	positive regulation of endopeptidase activity	0.01091039		
	release of cytochrome c from mitochondria	0.01105395		
	positive regulation of peptidase activity	0.01105395		

MODULE	TOP TERMS (Max 10)	Q VAL	GENES	TERMS
M1	protein O-linked fucosylation	0.00227777	85	39
	Golgi to lysosome transport	0.00333888		
	negative regulation of microtubule polymerization	0.00635436		
	protein O-linked glycosylation	0.00635436		
	Golgi to vacuole transport	0.00902354		
	cellular response to hypoxia	0.00909457		
	fucosylation	0.00909457		
	cellular response to decreased oxygen levels	0.00942207		
	cellular response to estrogen stimulus	0.00942975		
	cellular response to oxygen level	0.01050406		
M2	positive regulation of translation	0.00375400	30	17
	positive regulation of cellular amide metabolic process	0.00608075		
	positive regulation of intrinsic apoptotic signaling pathway	0.00680254		
	cellular response to hydrogen peroxide	0.00775096		
	response to hydrogen peroxide	0.01142272		
	cellular response to antibiotic	0.01190409		
	positive regulation of NF-kappaB transcription factor activity	0.01256329		
	cytoplasmic translation	0.01358535		
	positive regulation of cysteine-type endopeptidase activity	0.01375108		
	cellular response to reactive oxygen species	0.01408631		
M3	nucleoside metabolic process	0.00107571	97	84
	attachment of spindle microtubules to kinetochore	0.00107571		
	attachment of mitotic spindle microtubules to kinetochore	0.00107571		
	urate biosynthetic process	0.00107571		
	glycosyl compound metabolic process	0.00138042		
	ribonucleoside metabolic process	0.00343995		
	mitotic metaphase plate congression	0.00567666		
	urate metabolic process	0.00638538		
	pyrimidine ribonucleotide biosynthetic process	0.00659361		
	ribosomal small subunit export from nucleus	0.00680254		
M4	nucleosome organization	0.00333888	25	6
	androgen receptor signaling pathway	0.00519331		
	nucleosome assembly	0.00680254		
	intracellular steroid hormone receptor signaling pathway	0.01019257		
	steroid hormone-mediated signaling pathway	0.01142272		
	hormone-mediated signaling pathway	0.01393868		

M5	endoplasmic reticulum tubular network organization<br>establishment of protein localization to endoplasmic reticulum<br>positive regulation of actin filament polymerization<br>regulation of mitotic spindle organization<br>peptidyl-lysine hydroxylation<br>regulation of spindle organization<br>positive regulation of protein polymerization<br>vesicle targeting, to, from or within Golgi<br>regulation of actin filament polymerization<br>protein localization to endoplasmic reticulum	0.00107571<br>0.00107571<br>0.00362713<br>0.00430481<br>0.00430481<br>0.00567666<br>0.00635436<br>0.00659361<br>0.00680254<br>0.00693605	100	73
M6	mitotic spindle assembly<br>spindle assembly<br>mitotic spindle organization	0.00333888<br>0.00635436<br>0.00680254	26	29

Table S4. List of the enriched GO terms for biological functions relative to SuExt-ethosomes-treated cells *vs.* ethosomes-treated cells.

MODULE	TOP TERMS (Max 10)	Q VAL	GENES	TERMS
M1	viral gene expression	0.00102238	91	79
	mitotic cytokinesis	0.00186748		
	mRNA 5'-splice site recognition	0.00324434		
	IRES-dependent viral translational initiation	0.00392052		
	nuclear-transcribed mRNA catabolic process, nonsensemediated	0.00485962		
	viral translation	0.00561024		
	positive regulation by host of viral transcription	0.00604476		
	positive regulation of DNA binding	0.00651397		
	mRNA cis splicing, via spliceosome	0.00651397		
	positive regulation of viral transcription	0.00794067		
M2	negative regulation of binding	0.00002951	175	271
	positive regulation of epithelial to mesenchymal transition	0.00042650		
	positive regulation of pseudopodium assembly	0.00086672		
	regulation of pseudopodium assembly	0.00102238		
	viral entry into host cell	0.00134844		
	transforming growth factor beta receptor signaling pathway	0.00134844		
	entry into host cell	0.00134844		
	entry into host	0.00134844		
	entry into cell of other organism involved in symbiotic interaction	0.00134844		
	entry into other organism involved in symbiotic interaction	0.00134844		
M3	regulation of release of cytochrome c from mitochondria	0.00216082	87	53
	release of cytochrome c from mitochondria	0.00369617		
	negative regulation of intrinsic apoptotic signaling pathway in response to DNA damage by p53 class mediator	0.00535170		
	negative regulation of release of cytochrome c from mitochondria	0.00604476		
	regulation of oxidoreductase activity	0.00752686		
	regulation of intrinsic apoptotic signaling pathway in response to DNA damage by p53 class mediator	0.00800700		
	regulation of signal transduction by p53 class mediator	0.00809619		
	regulation of viral genome replication	0.00809619		
	apoptotic mitochondrial changes	0.00946896		
	negative regulation of intrinsic apoptotic signaling pathway by p53 class mediator	0.00984061		
M4	cardiac muscle hypertrophy	0.00754405	131	38
	striated muscle hypertrophy	0.00794067		
	muscle hypertrophy	0.00800700		
	positive regulation of cardiac muscle hypertrophy	0.00809619		
	positive regulation of muscle hypertrophy	0.00837906		
	response to muscle stretch	0.01006818		
	negative regulation of type I interferon production	0.01535191		
	gene silencing by miRNA	0.01839324		

	regulation of cardiac muscle hypertrophy	0.01912719		
	cytoplasmic mRNA processing body assembly	0.02073874		
M5	protein monoubiquitination	0.00466278	179	65
	regulation of mitochondrial membrane permeability	0.00800700		
	sister chromatid biorientation	0.00935982		
	regulation of membrane permeability	0.00949099		
	attachment of mitotic spindle microtubules to kinetochore	0.01222483		
	histone ubiquitination	0.01379838		
	histone H4-K16 acetylation	0.01626329		
	positive regulation of mitochondrial membrane permeability involved in apoptotic process	0.01952702		
	mitochondrial outer membrane permeabilization involved in programmed cell death	0.02038969		
	histone H2A ubiquitination	0.02038969		
M6	positive regulation of response to DNA damage stimulus	0.01321329	30	7
	regulation of receptor-mediated endocytosis	0.01448120		
	regulation of proteasomal ubiquitin-dependent protein catabolic	0.01950925		
	DNA-templated transcription, initiation	0.01950925		
	receptor metabolic process	0.02209452		
	regulation of ubiquitin-dependent protein catabolic process	0.02256698		
	regulation of proteasomal protein catabolic process	0.02515290		
**MODULE**	**TOP TERMS (Max 10)**	**Q VAL**	**GENES**	**TERMS**
M1	cytoplasmic translation	0.00002259	58	43
	viral translation	0.00206521		
	negative regulation of cell junction assembly	0.00264358		
	protein N-linked glycosylation	0.00287829		
	negative regulation of peptidyl-serine phosphorylation	0.00470643		
	cellular response to antibiotic	0.00476779		
	positive regulation of translation	0.00767814		
	positive regulation of apoptotic signaling pathway	0.00871464		
	negative regulation of protein kinase B signaling	0.00947006		
	viral gene expression	0.01199265		
M2	telomere maintenance via telomerase	0.00002397	126	170
	telomere maintenance via telomere lengthening	0.00004750		
	regulation of DNA biosynthetic process	0.00006795		
	RNA-dependent DNA biosynthetic process	0.00006795		
	positive regulation of DNA biosynthetic process	0.00008459		
	positive regulation of telomerase activity	0.00011632		
	diadenosine polyphosphate biosynthetic process	0.00024354		
	diadenosine tetraphosphate biosynthetic process	0.00024354		
	regulation of telomerase activity	0.00042916		
	mRNA export from nucleus	0.00042990		

M3	cellular response to oxygen levels	0.00490165	121	54
	activation of cysteine-type endopeptidase activity	0.00606272		
	cellular response to platelet-derived growth factor stimulus	0.00606272		
	response to platelet-derived growth factor	0.00720784		
	endosomal vesicle fusion	0.00749196		
	execution phase of apoptosis	0.00749606		
	endoplasmic reticulum tubular network organization	0.00827682		
	cytosolic transport	0.00979388		
	endoplasmic reticulum organization	0.01100892		
	retrograde transport, endosome to Golgi	0.01131784		
M4	mitochondrial respiratory chain complex I assembly	0.00038528	116	71
	NADH dehydrogenase complex assembly	0.00039795		
	mitochondrial respiratory chain complex assembly	0.00068216		
	proline biosynthetic process	0.00173223		
	GDP-mannose metabolic process	0.00206521		
	establishment of protein localization to chromatin	0.00285823		
	proline metabolic process	0.00476779		
	mRNA polyadenylation	0.00476779		
	nucleotide-sugar biosynthetic process	0.00514115		
	RNA polyadenylation	0.00631821		
M5	cellular response to increased oxygen levels	0.00002265	165	284
	fatty acid derivative metabolic process	0.00004750		
	icosanoid metabolic process	0.00009168		
	unsaturated fatty acid metabolic process	0.00010199		
	response to increased oxygen levels	0.00011037		
	polyketide metabolic process	0.00011068		
	aminoglycoside antibiotic metabolic process	0.00011068		
	daunorubicin metabolic process	0.00011068		
	doxorubicin metabolic process	0.00011068		
	xenobiotic catabolic process	0.00020331		
M6	calcium import into the mitochondrion	0.00117796	39	10
	mitochondrial transmembrane transport	0.00229905		
	mitochondrial calcium ion transmembrane transport	0.00289891		
	mitochondrial calcium ion homeostasis	0.00342494		
	calcium ion import	0.01099604		
	protein tetramerization	0.01109682		
	protein localization to mitochondrion	0.01942961		
	mitochondrial membrane organization	0.02076735		
	nucleoside diphosphate metabolic process	0.02088002		
	cellular response to radiation	0.03453784		
M7	COPII-coated vesicle budding	0.00002259	51	24
	Golgi vesicle budding	0.00002397		
	vesicle budding from membrane	0.00006795		
	cargo loading into COPII-coated vesicle	0.00006795		

	cargo loading into vesicle	0.00011117		
	ER to Golgi vesicle-mediated transport	0.00062594		
	endoplasmic reticulum tubular network organization	0.00296872		
	positive regulation of viral genome replication	0.00476779		
	positive regulation of Wnt signaling pathway	0.00792428		
	positive regulation of viral life cycle	0.00947006		
M8	cytosolic transport	0.00730879	11	1

Table S5. List of the enriched GO terms for biological functions relative to SuExt-ethosomes-treated cells *vs.* sumac extract-treated cells.

MODULE	TOP TERMS (Max 10)	Q VAL	GENES	TERMS
M1	negative regulation of endothelial cell migration	0.00071816	82	173
	regulation of endothelial cell migration	0.00126055		
	negative regulation of epithelial cell migration	0.00126055		
	regulation of cell adhesion molecule production	0.00139441		
	negative regulation of blood vessel endothelial cell migration	0.00139441		
	positive regulation of cholesterol esterification	0.00178677		
	cell adhesion molecule production	0.00178677		
	regulation of cholesterol esterification	0.00200861		
	regulation of Cdc42 protein signal transduction	0.00200861		
	negative regulation of heterotypic cell-cell adhesion	0.00200861		
M2	fibrinolysis	0.00071816	83	89
	positive regulation of blood coagulation	0.00126055		
	positive regulation of hemostasis	0.00126055		
	positive regulation of coagulation	0.00139441		
	negative regulation of fibrinolysis	0.00171352		
	negative regulation of blood coagulation	0.00178677		
	negative regulation of coagulation	0.00178677		
	negative regulation of hemostasis	0.00178677		
	regulation of fibrinolysis	0.00200861		
	blood coagulation	0.00310636		
M3	mitochondrial membrane organization	0.00200861	76	54
	negative regulation of cell cycle arrest	0.00347731		
	cell cycle arrest	0.00387897		
	protein homotrimerization	0.00396029		
	positive regulation of release of cytochrome c from mitochondria	0.00500788		
	cristae formation	0.00611335		
	protein trimerization	0.00706087		
	electron transport chain	0.00737375		
	positive regulation of cysteine-type endopeptidase activity	0.00843431		
	regulation of release of cytochrome c from mitochondria	0.00898663		
M4	regulation of lipid transport by regulation of transcription from RNA polymerase II promoter	0.00139441	119	80
	signal transduction involved in mitotic cell cycle checkpoint	0.00451436		
	signal transduction involved in mitotic DNA damage checkpoint	0.00451436		
	signal transduction involved in mitotic DNA integrity checkpoint	0.00451436		
	negative regulation of TOR signaling	0.00475127		
	mitotic DNA damage checkpoint	0.00485066		
	phosphatidylinositol-3-phosphate biosynthetic process	0.00567166		
	mitotic DNA integrity checkpoint	0.00706087		
	signal transduction involved in DNA integrity checkpoint	0.00837117		

MODULE	TOP TERMS (Max 10)	Q VAL	GENES	TERMS
	signal transduction involved in DNA damage checkpoint	0.00837117		
M1	cytoplasmic translation	0.00031829	36	13
	positive regulation of translation	0.00473875		
	positive regulation of cellular amide metabolic process	0.00624152		
	positive regulation of intrinsic apoptotic signaling pathway	0.00702949		
	cellular response to hydrogen peroxide	0.00854761		
	response to hydrogen peroxide	0.01263497		
	cellular response to antibiotic	0.01369615		
	positive regulation of cysteine-type endopeptidase activity involved in apoptotic process	0.01511182		
	cellular response to reactive oxygen species	0.01511182		
	positive regulation of apoptotic signaling pathway	0.01733998		
M2	telomerase holoenzyme complex assembly	0.00168803	93	81
	maintenance of DNA methylation	0.00326836		
	nucleoside metabolic process	0.00340957		
	protein localization to mitochondrion	0.00349334		
	glycosyl compound metabolic process	0.00524012		
	AMP biosynthetic process	0.00536995		
	pyrimidine ribonucleotide biosynthetic process	0.00583207		
	chaperone-mediated protein complex assembly	0.00624152		
	ribosomal small subunit export from nucleus	0.00624152		
	pyrimidine ribonucleoside biosynthetic process	0.00678798		
M3	positive regulation of intracellular protein transport	0.01511182	46	4
	interaction with host	0.01710998		
	positive regulation of intracellular transport	0.02520181		
	regulation of intracellular protein transport	0.02740930		
M4	hormone-mediated signaling pathway	0.00536995	8	1
M5	COPII-coated vesicle budding	0.00122121	92	85
	positive regulation of myeloid cell differentiation	0.00164402		
	Golgi vesicle budding	0.00164402		
	vesicle budding from membrane	0.00458250		
	cargo loading into COPII-coated vesicle	0.00536995		
	positive regulation of endothelial cell proliferation	0.00544563		
	vesicle targeting, to, from or within Golgi	0.00544563		
	regulation of actin polymerization or depolymerization	0.00624152		
	regulation of actin filament length	0.00624152		
	regulation of myeloid cell differentiation	0.00702949		
M6	cellular modified amino acid metabolic process	0.00490127	6	1
M7	establishment of monopolar cell polarity	0.00017441	3	5
	establishment of apical/basal cell polarity	0.00017441		
	establishment or maintenance of monopolar cell polarity	0.00017441		
	establishment or maintenance of apical/basal cell polarity	0.00044219		
	establishment or maintenance of bipolar cell polarity	0.00044219		
